# In-situ interfacial compatibilization via edge-sulfurated few layer graphene during the formation of crosslinked graphene-rubber nanocomposites

**DOI:** 10.1038/s41598-022-08071-w

**Published:** 2022-03-07

**Authors:** Sangeeth Krishnan, Maya M.G, Akhil Das, Suja Bhargavan, Krithika Ganesan, Swaminathan Sivaram, Prabha Vadivelu, Lakshminarayanan Ragupathy

**Affiliations:** 1 Corporate R&D Center, HLL Lifecare Limited, Akkulam, Sreekariam (P.O), Thiruvananthapuram, 695017 India; 2 Deapartment of Chemistry, Central University of Tamil Nadu, Neelakudi, Thiruvarur, 610005 India; 3 Indian Institute of Science Education and Research, Dr. Homi Bhabha Road, Pune, 411008 India

**Keywords:** Graphene, Nanoscale materials, Techniques and instrumentation

## Abstract

Herein, we report various physico-chemical approaches to probe the nature of the interface between few layers graphene (FLG) and carboxylated nitrile rubber (XNBR) nanocomposites prepared through efficient blending of XNBR latex with an aqueous dispersion of FLG. The extent of physical interaction between FLG and XNBR was investigated using Lorentz–Park and Cunneen–Russell models. The chemical interface between FLG and sulfur crosslinked XNBR was studied using model reactions between sulfur and graphene in presence of zinc 2-mercaptobenzothiazole (ZMBT). We propose that an edge sulfurated FLG is formed, which could chemically bond with XNBR during the vulcanization process. Density Functional Theory (DFT) was employed to unravel the mechanistic insights, which support this hypothesis and suggest a kinetically favorable sulfuration of both XNBR and FLG. The formation of a chemical bond between edge-FLG and XNBR through the proposed intermediacy of sulfurated FLG leads to the observed improvement in mechanical properties of the nanocomposites.

## Introduction

Nano or micro sized filler materials such as carbon black, silica, montmorillonite, carbon nanotubes and graphene are commonly used as reinforcing agents to improve the physical and mechanical properties of the elastomers^1,2^. Among the fillers employed in rubbers, graphene is exciting because of its huge specific surface area (2600 m^2^ /g), and unexpected mechanical, electrical, thermal as well as gas barrier properties^3–8^. Literature reports many studies on graphene-rubber nanocomposites^9–19^. However, inadequate dispersion of graphene in the rubber matrix due to poor interfacial interaction between graphene and elastomers continues to be a challenge^4^. While the nature of mixing techniques used is important, the uniformity of dispersion of the FLG in the rubber matrix will require favorable interfacial interaction through covalent and non-covalent bonds^11–14^.

The frequently employed strategy to increase the interfacial interaction between graphene and a polymer is surface modification of graphene oxide (GO) or reduced GO (rGO) using different functional small molecules viz*.* organic isocyanates^20^, alkyl-chlorosilanes^21^, bis(triethoxysilylpropyl)tetrasulfide, amino acids^22^ and perylene^23^. Also, few polymers^24^ such as conjugated-polyelectrolyte^25^ and poly(vinyl alcohol)^26^ have been employed to modify GO. Styrene-butadiene rubber (SBR)-graphene nanocomposite was prepared using modified GO with alkylamines^27^, which significantly improved their interfacial interaction through sulfur and the double bond present in the alkylamines or through the use of orthoquinone-thiol chemistry^28^. More recently FLG has been successfully used to prepare thin film nanocomposites with rubbers available in the latex form such as natural rubber and XNBR (carboxylated nitrile rubber)^29–31^. A good balance of tensile and elongational property has been reported. Unlike r-GO, FLG is easy to produce with little or no defects using shear induced exfoliation of graphite. Furthermore, FLG has no functional groups capable of interacting with rubbers, thus, the nature of chemical and physical interactions between FLG and crosslinked rubber still remains a mystery.

In this paper, we report a combination of experimental and computational evidence to define the nature of interface between FLG and carboxylated nitrile rubber (XNBR). The degree of non-covalent interaction between XNBR and FLG in XNBR-FLG nanocomposites, was established using swelling experiments through Lorentz-Park and Cunneen-Russell equations. Model reactions were performed between FLG, S, ZMBT and ZnO (except XNBR) and the resultant product had been purified and characterized. Based on these results and the computational studies using DFT, we propose a novel chemical hypothesis for the interaction between FLG and XNBR.

## Results and discussions

FLG was produced using a planetary ball mill using reported protocol^30^. The presence of defect-free FLG was confirmed by Transmission Electron Microscopy (TEM), Raman Spectroscopy and X-ray Photoelectron Spectroscopy (XPS). Different amount of FLG was mixed with XNBR latex using a microfluidizer and thin films were produced using a film casting/dip molding method. The resulted thin film nanocomposites show the improvements in tensile properties up to 110% and the complete details will be published elsewhere^31^.

XNBR possesses three functionalities, namely, pendant nitrile, carboxylic acid groups and in-chain unsaturation since it is a random terpolymer of acrylonitrile, butadiene and acrylic/methacrylic acid. These three functional groups can contribute in crosslinking process by forming covalent and ionic bonds^32,33^. There has been a few published reports on the preparation and characterization of graphene-XNBR nanocomposites^23,34^. GO or its derivatives and a binary curing system comprising of MgO and sulfur/peroxide were typically employed. It is believed that the chemical functionalities present in GO/rGO react with the functionalities on the XNBR leading to a chemical bond between graphene and polymer, thus, creating a strong polymer–graphene interface that leads to the observed improvement in properties. On the contrary, FLG is devoid of any chemical functionality. Therefore, it was surprising to observe that FLG also causes significant improvements in mechanical properties when FLG is mixed with XNBR even without a putative mechanism for the formation of chemical bond between FLG and XNBR.

### Crosslinking density of XNBR-FLG nanocomposites

**Figure 1:**
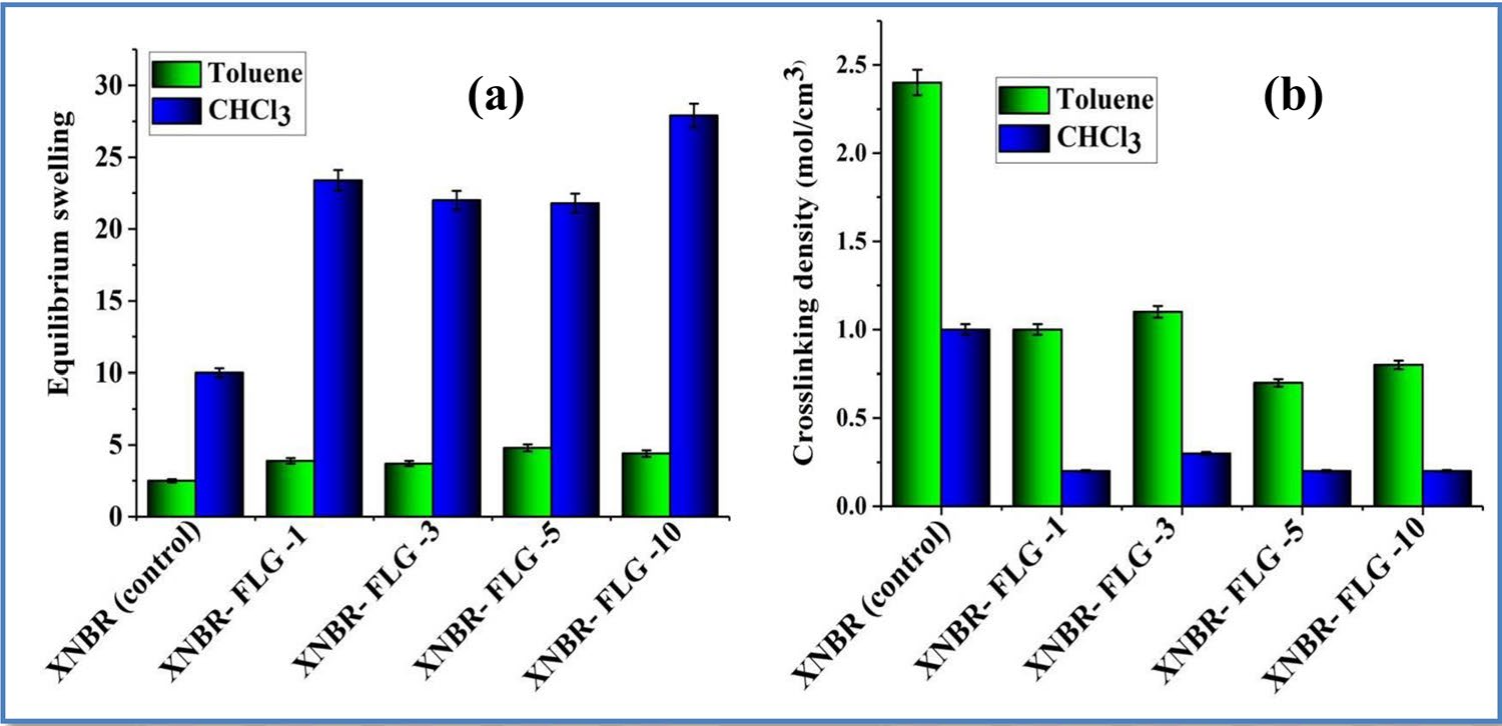
Swelling studies on FLG incorporated XNBR nanocomposite thin films in toluene and chloroform; (**a**) equilibrium swelling (**b**) crosslinking density. The numbers (1, 3, 5 and 10) in X axis indicates wt% of the FLG with respect to rubber.

Equilibrium swelling ratio of thin film FLG-XNBR nanocomposites prepared by microfluidizer was investigated using two solvents viz*.* toluene and chloroform (SI-Sect. 1). All the films attain equilibrium swelling within 24 h of solvent uptake^35^. The equilibrium swelling ratio of the nanocomposite is observed to be higher (i.e. 2–3 times) in chloroform compared to toluene (Fig. 1). This could be due to the lower interaction parameter (χ = 0.34) of XNBR-chloroform (i.e. higher thermodynamic interaction) compared to XNBR-toluene (χ = 0.41) (see SI-Sect. 1). Equilibrium swelling is significantly higher for FLG incorporated XNBR nanocomposites compared to XNBR in both solvents. Accordingly, compared to XNBR, FLG-XNBR nanocomposites show lower crosslinking density, which was calculated using Flory-Rehner equation [SI-(3)]. Crosslinking density calculated from equilibrium swelling method is the sum total of chemical crosslinks between the polymer, entanglements of macromolecular chain, and the polymer chain–filler networks^36^. In the absence of any functional groups, FLG is incapable of forming chemical bonds with the rubber. Consequently, FLG-XNBR nanocomposites show a reduced crosslinking density compared to XNBR. Notwithstanding the lower degree of crosslinking, the nanocomposites show improved tensile properties without adversely affecting either the modulus or elongation properties^31^. This implies some favorable interaction between FLG and XNBR.

### Nature of physico-chemical interaction between FLG and XNBR

Covalent bond formation between GO/rGO and rubber via sulfur has been reported^37^. Unlike GO, FLG lacks any reactive functional group, therefore, is expected to interact with XNBR through van der-Waals, π–π and hydrophobic interactions. The extent of non-covalent interaction between rubber and filler was estimated theoretically using Lorentz-Park^38^ and Cunneen-Russell equations^39^ by exploiting the swelling studies (SI-Sect. 1).

**Figure 2:**
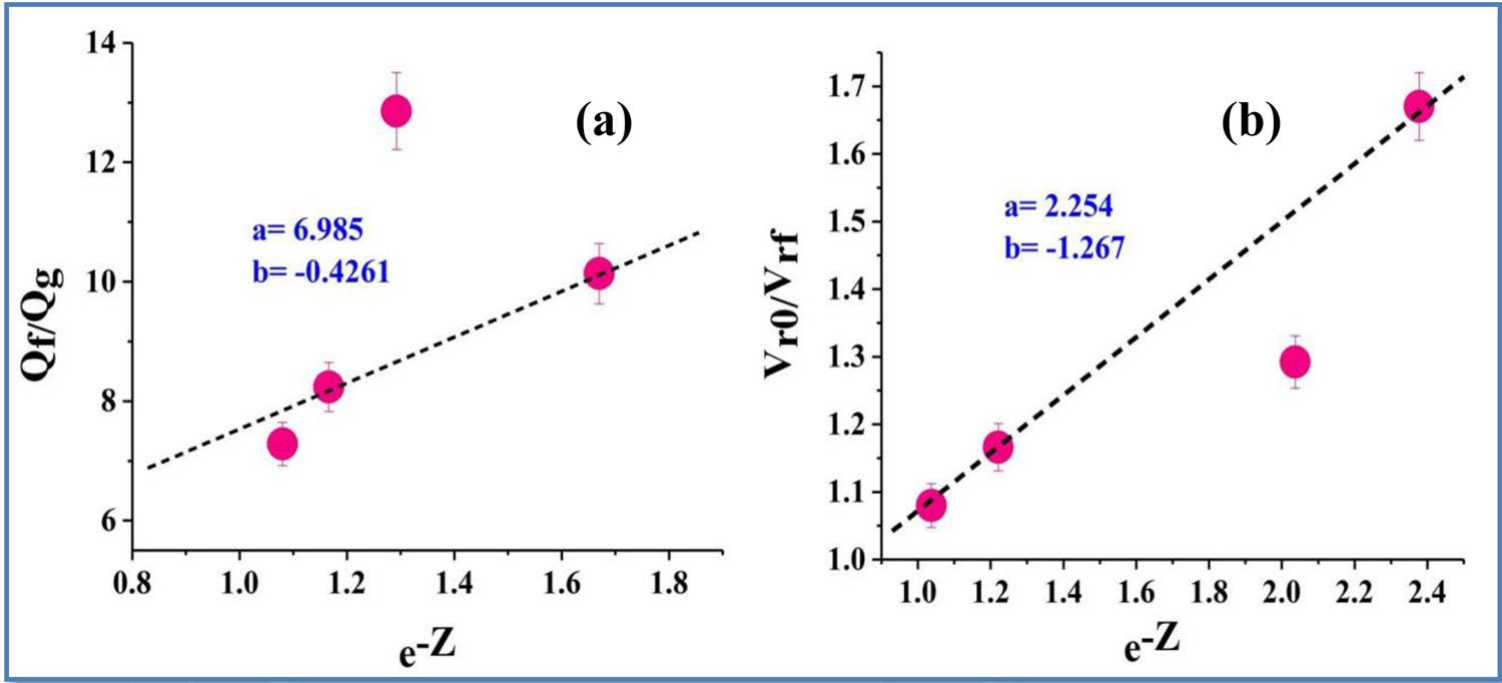
(**a**) Lorenz-Park fitting and (**b**) Cunneen-Russell plot of XNBR-FLG nanocomposites prepared by microfluidizer using chloroform as solvent.

The Lorentz-Park model^38^ (equation no.1) has been frequently employed to investigate the swelling of filler reinforced vulcanizates.

1 $$\frac{{\mathrm{Q}}_{f}}{{\mathrm{Q}}_{g}}=a{e}^{-z}+\mathrm{b}$$

where Q is quantity of the solvent per unit weight of the rubber (f and g indicate filled and gum blends, respectively). z is weight fraction of the filler whereas a and b are constants and depends on the filler behavior. The plot between ratio of quantity of solvent per unit weight of the filled rubber and free rubber ($\frac{Qf}{Qg}$) versus ${e}^{-Z}$ (z is the weight portion of filler) gives a straight line (Fig. 2a) with a positive slope (a = 6.99) and intercept (b = 0.43). This indicates that FLG has good reinforcing capability in XNBR even at low loadings.

The Cunneen-Russell equation (equation no.2) also accounts for the enhancement of crosslinking efficiency by the curing agents. As a result, the employed Cunnen-Russell equations^39^ signifies the ratio of volume fractions of the rubber in filled and unfilled systems instead of the amount of solvent absorbed per unit weight of the rubber.

2 $$\frac{{\mathrm{V}}_{ro}}{ {\mathrm{V}}_{rf}}=a{e}^{-z}+\mathrm{b}$$

where, z is weight fraction of the filler, a and b are constants which depends on the filler behavior. V_ro_ is volume fraction of the rubber gum vulcanizate, V_rf_ is volume fraction of the elastomer in the solvent swollen filled sample and is given by the equation of Ellis and Welding:

3 $${V}_{rf}=\frac{(d-fw)/{\uprho }_{P}}{(d-fw)/{\uprho }_{P}+{A}_{S}/{\uprho }_{S}}$$

where d is swollen weight, ƒ is volume fraction of the filler, w is initial weight of the sample, ρ_p_ is density of the polymer, ρ_s_ is density of the solvent, and A_s_ is amount of the solvent absorbed.

4 $${V}_{ro}=\frac{[{W}_{d}-{W}_{f}/{\uprho }_{d}]}{\left[{W}_{d}-{W}_{f}\right]/{\uprho }_{d}+{W}_{S}/{\uprho }_{s}}$$

where W_d_ and W_s_ are weight of the dry rubber and weight of the solvent absorbed by the sample, respectively. W_f_ is weight of the filler in the sample, ρ_d_ is density of the rubber compound, and ρ_s_ is density of toluene (0.867 g/cm^3^). Here, the plot between ratio of volume fractions of unfilled and filled rubber (V_r0_/V_rf_) and ${e}^{-Z}$ yields a straight line (Fig. 2b). A combination of higher value of a (2.25) with lower value of b (− 1.27) suggests a strong interaction between FLG and XNBR. In both the graphs (Fig. 2), one point is observed to be extremely outlined. The scientific reasons for this behavior are not understood at the present time. However, the similar behavior was also observed elsewhere^40,41^.

Interfacial interaction (either covalent or non-covalent or both) between filler and polymer is responsible for the property improvement. The non-covalent interactions, however, are weaker compared to covalent interaction and generally not capable of giving large improvements in mechanical properties. To achieve a complete benefit of graphene in the reinforcement of elastomers, it is essential to increase the interfacial interaction, which can be maximized by the covalent bonds. Incorporation of GO/functionalized GO in rubber has increased the crosslinking density (covalent bond formation between oxygen functionals and rubber), which results improvement in the mechanical properties. To explain the substantial increase in tensile strength observed with the FLG-XNBR nanocomposites prepared in this study, we propose the existence of a covalent interaction between XNBR and FLG through its edges via di-sulfide bond formation. At the same time, it is difficult to establish the formation of FLG-XNBR bond experimentally due to the system complexity. Therefore, a model reaction was performed (without XNBR) between sulfur, ZnO and zinc 2-mercaptobenzothiazole (ZMBT) and FLG in water at 70 and 100 °C for 24 h. The product of this reaction was purified by washing with hot water (to remove melamine) followed by Soxhlet extraction in CHCl_3_ (to remove un-reacted sulfur and ZMBT) and subjected to XPS analysis.

**Figure 3:**
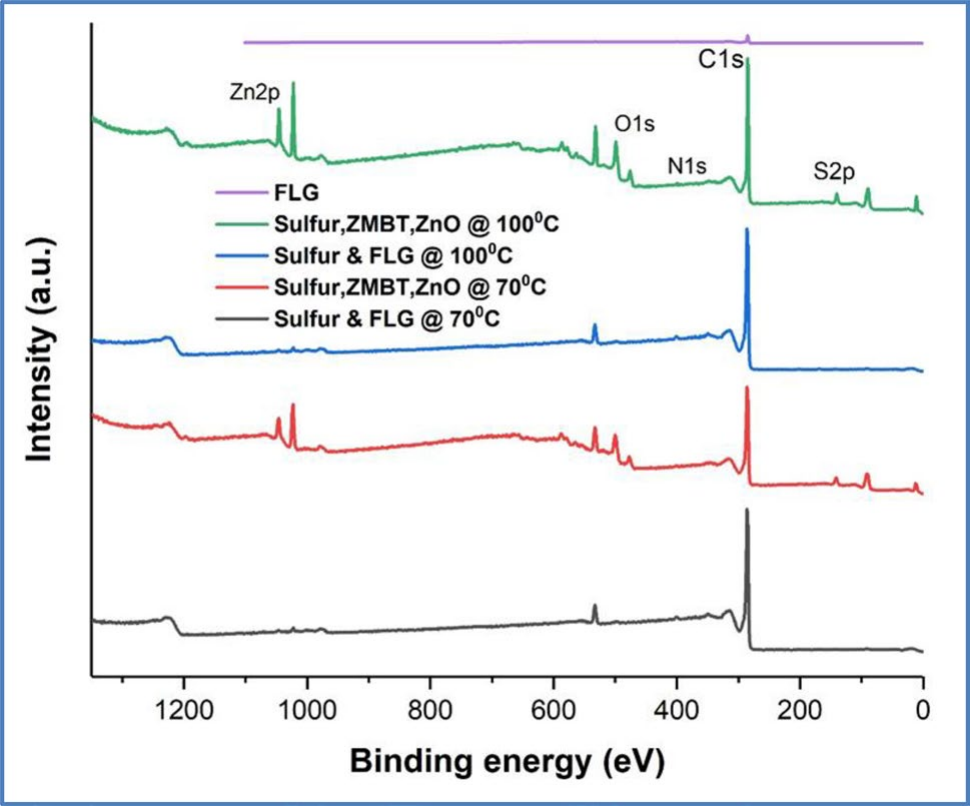
XPS survey spectra for FLG, control experiments (Sulfur and FLG) performed at 70 and 100 °C and the purified products from the model reactions.

**Figure 4:**
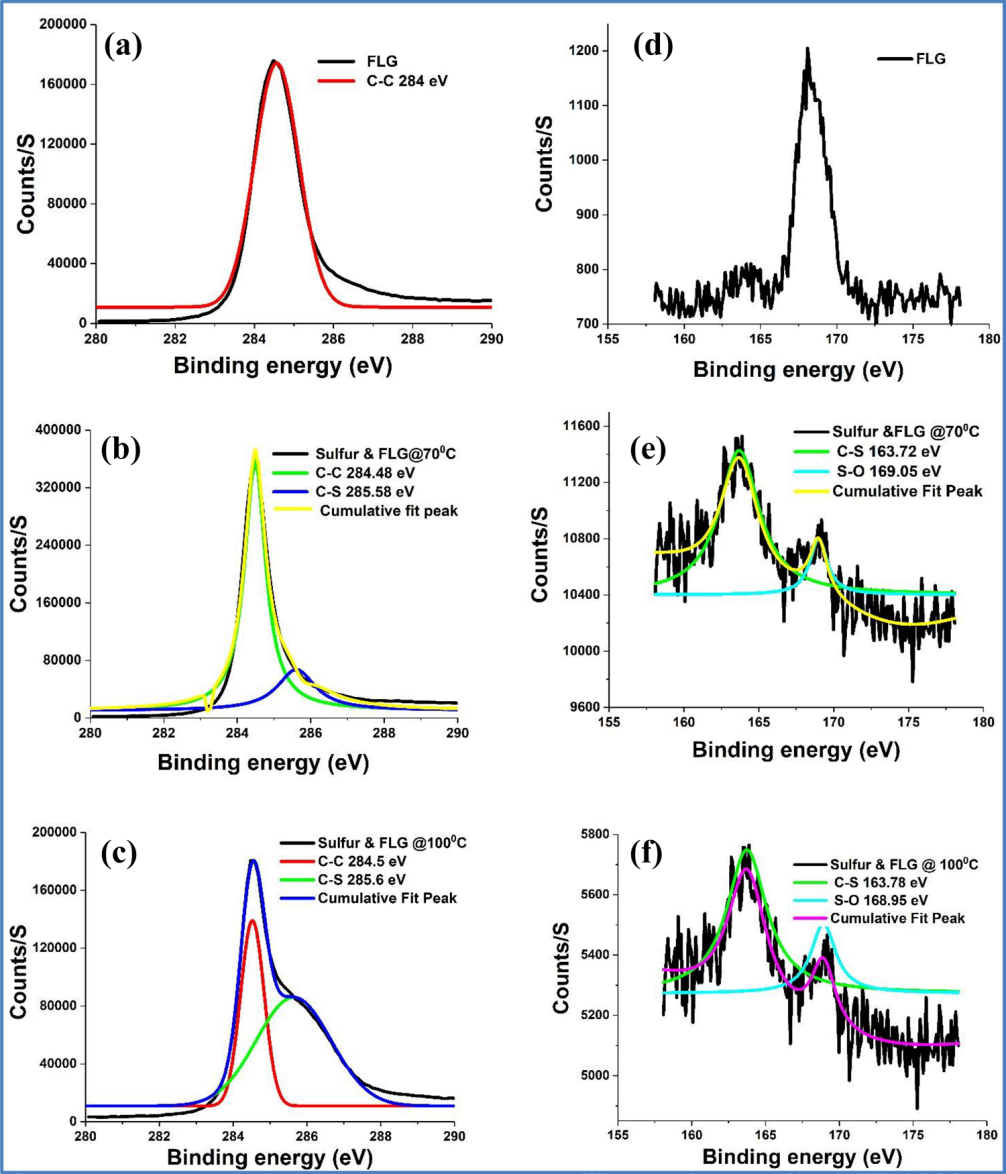
XPS spectra for C1s for (**a**) FLG (**b**) control experiments (FLG and sulfur) performed at 70 °C and (**c**) 100 °C. S2p spectra for (**d**) FLG and (**e**) control experiments (FLG and sulfur) performed at 70 °C and (**f**) 100 °C.

**Figure 5:**
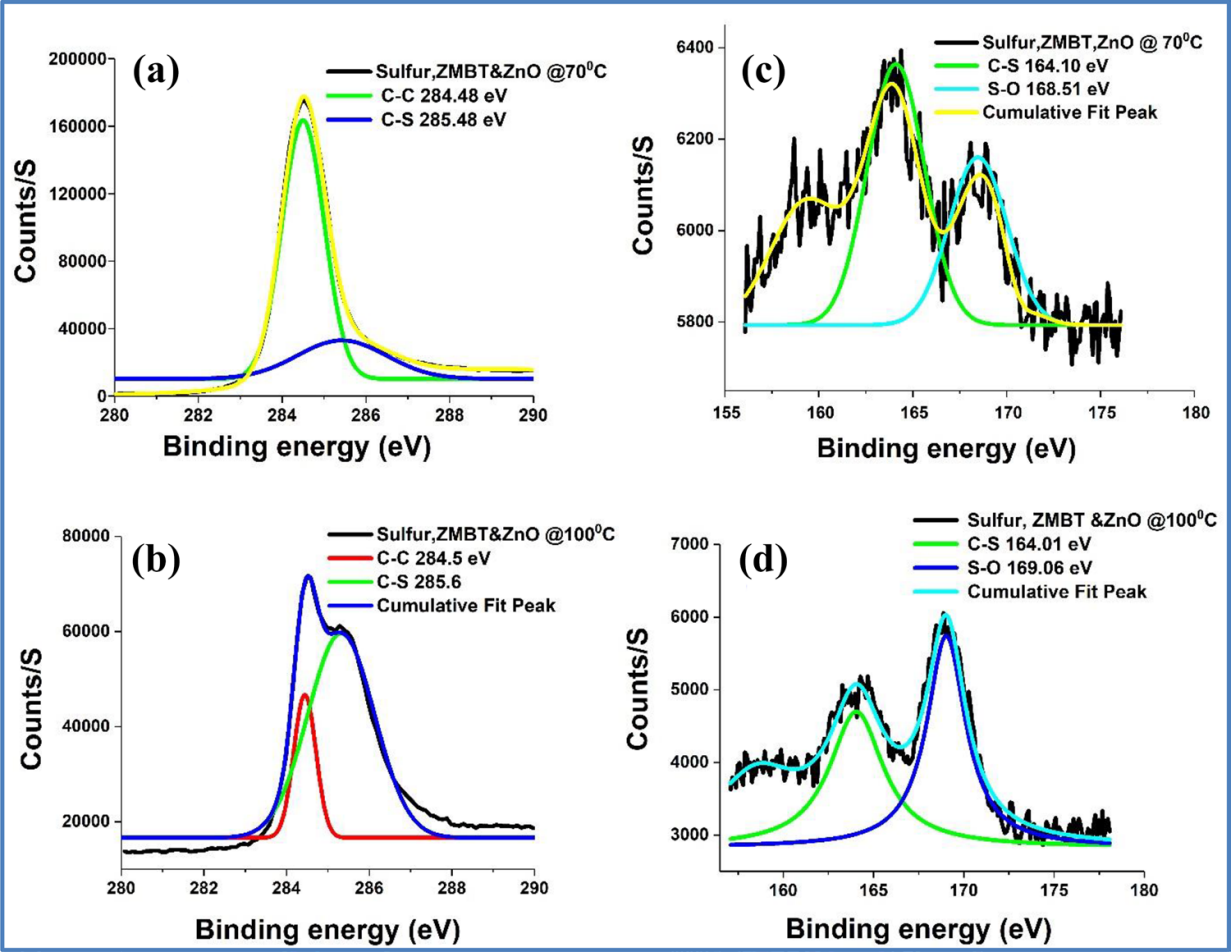
XPS spectra for C1s for sulfurated FLG prepared (**a**) at 70 °C and (**b**) 100 °C. S2p spectra for sulfurated FLG prepared (**c**) at 70 °C and (**d**) 100 °C.

XPS survey spectra of all the investigated samples are given in Fig. 3. Figure 4a–c and 5a,b show the high resolution XPS spectra for C1s core level peak of FLG, control experiments performed at 70 and 100 °C and the purified products from the model reactions, respectively. Here, the main C1s peak is observed at 284 eV, suggesting that most of the C atoms remained in the conjugated honeycomb lattice, like sp^2^ C in the graphite. Compared to the pure FLG and the products from the control experiments, both the products of the model reactions [5(a)&(b)] have broadened C 1 s peak due to the mixture of sp^2^ C and C–S atoms with high binding energy (285 eV), suggesting the presence of sulfurated FLG. These observations are in good accordance with the structure of S-doped diamond and S modified SWCNTs^42,43^.

Figures 4d–f and 5c,d show the high resolution XPS spectra for S 2p core level peaks of FLG, control experiments and sulfurated FLGs, respectively. The calculated atomic % of sulfur in sulfurated FLG prepared at 70 and 100 °C is 0.4 and 3.6, respectively. High resolution S 2p XPS spectra of sulfurated graphene prepared at 70 °C showed a broad and intense peak at 163.5 eV and a less intense peak at 169 eV. However, the later peak becomes significant in the case of sulfurated FLG prepared at 100 °C. Sulfur binding energy of 163.5 eV for C-S compounds implies no significant charge separation.

To confirm that the observed S 2p peak is due to sulfurated FLG and not because of impurities, UV analysis of these samples were performed in CHCl_3_ [Figure S1 and S2 & Table S3 and S4]. A calibration curve was made with different amount of ZMBT in CHCl_3_. UV analysis shows that 99.96% of ZMBT was removed after soxhlet extraction in the case of sulfurated FLG prepared at 70 °C. Residual 0.04% (represents 0.013 sulfur atomic wt%) is too small for detection by XPS. Based on these results, we conclude that the observed 0.4 atomic wt% of sulfur is largely on account of sulfurated FLG.

**Figure 6:**
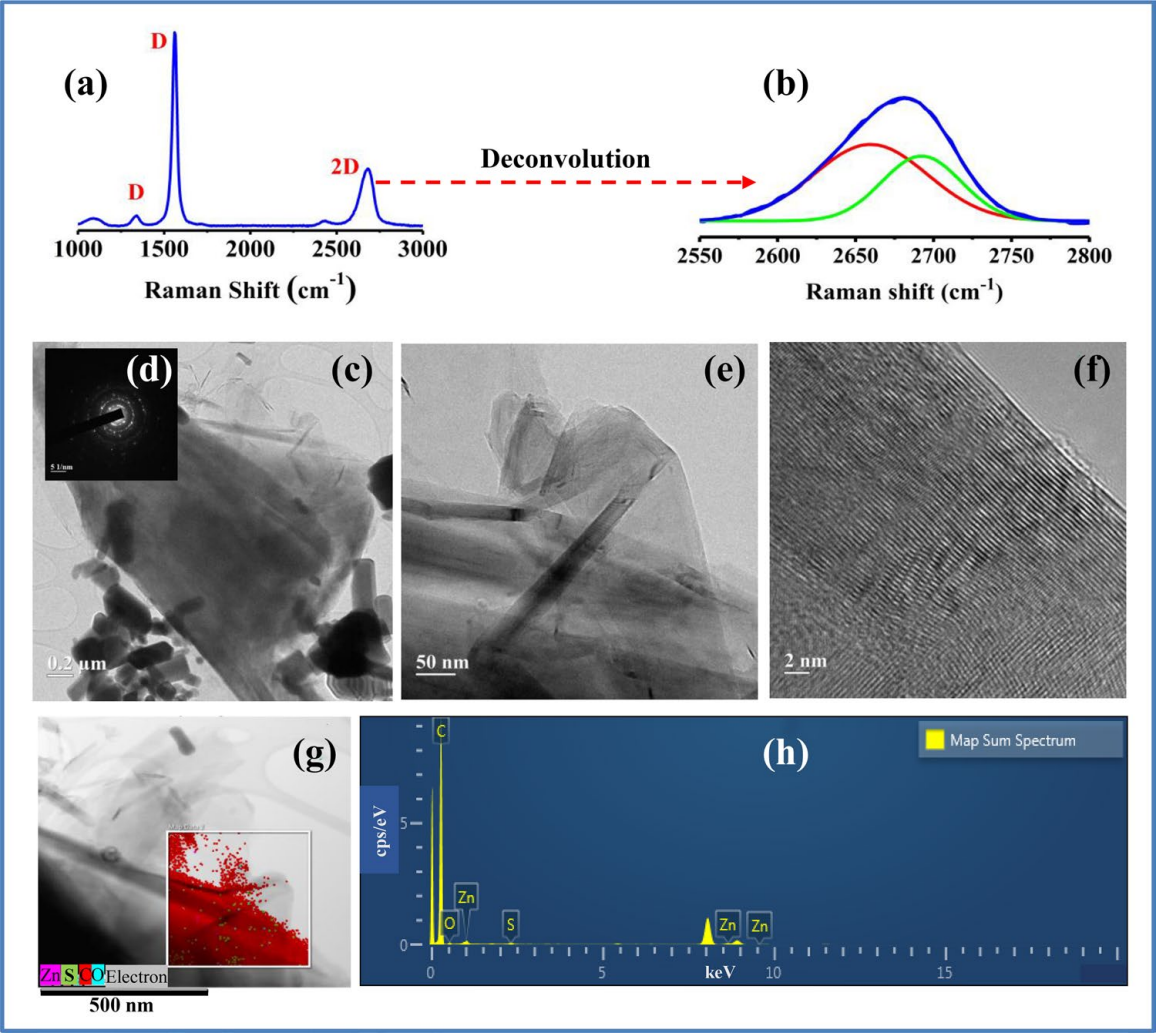
Characterization of sulfurated FLG prepared at 70 °C. Raman spectra (**a**) with deconvolution of 2D band (**b**) of sulfurated FLG. TEM images showing fringes of few layer sulfurated graphene (**c**–**f**). TEM with energy dispersive X-ray spectroscopy (TEM-EDS) image of sulfurated FLG (**g**,**h**).

The sulfurated FLG sample prepared at 70 °C was examined by Raman spectroscopy [Fig. 6a]. Raman spectra shows sharp and intense G band and 2D bands at 1560 and 2682 cm^−1^ respectively. Also, the sample shows an additional small Raman peak at around 1341 cm^−1^ as D band. The deconvolution of the 2D band leads to two Lorentzian peaks [Fig. 6b], which indicates that the sulfurated FLG is five layered^44,45^. TEM images clearly show the presence of sheet like structure along with ZnO crystals [Fig. 6c,e]. The multiple set of diffraction in SAED pattern [Fig. 6d] suggest the presence of FLG^46,47^. In addition, the graphene fringes correspond to multi layered graphene is clearly visible from [Fig. 6f]. TEM-EDS images of the sample [Fig. 6g] show the presence of 99.3, 0.5, 0.2 and 0.1 atomic % of carbon, oxygen, sulfur and zinc, respectively [Fig. 6h].

### DFT modeling of the mechanism of ZMBT assisted sulfuration on FLG and XNBR

To better understand the experimental findings, the DFT study was undertaken (Section S3). The sulfur crosslinking mechanism is initiated by the sulfuration of FLG or XNBR, both subsequently link together to form the sulfur crosslinked FLG-XNBR nanocomposite. Pyrene and 2-pentene were employed as model compounds to mimic the reactivity of FLG and an unsaturated rubber. Nieuwenhuizen et al.^48^ have previously reported bis(dimethyldithiocarbamato)zinc(II) (ZDMC) mediated sulfuration of natural rubber using experimental and computational methods. Due to the difficulty in finding out the transition states, the authors approximately estimated the activation barrier (+ 21.5 kcal/mol) for the ZDMC mediated sulfuration of natural rubber (propene as a model compound) using restricted optimization in transition state geometries.

**Figure 7:**
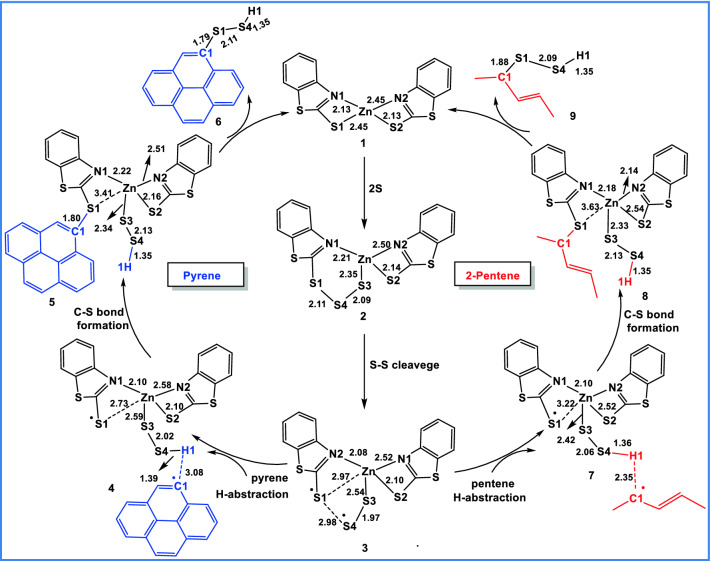
Modelled mechanism for ZMBT assisted sulfuration of pyrene and 2-pentene. Optimized bond lengths are shown in Å.

Based on Nieuwenhuizen et al. findings^48^, we have modelled the following mechanistic pathways (Fig. 7) for ZMBT mediated sulfuration of pyrene. In the first step, the di-sulfide (S_2_ from the sulfur source) group is incorporated into the ZMBT (**1**) to generate the intermediate ZMBT-2S (**2**). In the next step, one of the S–S bonds in **2** dissociates leading to the formation of sulfur radicals as shown in ZMBT-2S_rad_ (**3**). The sulfur radicals in **3** activate the C–H bond of pyrene, via H-atom abstraction and formation of C–S bond. In the H-atom abstraction step, one of the H-atoms in pyrene is abstracted by the sulfur radical in **3**, leading to the formation of **4**. This subsequently undergoes the C–S bond formation to yield the sulfur incorporated pyrene (**5**). Further, the S–S bond formation in **5** yields pyrene-S–S–H (**6**) and regenerates ZMBT (**1**).

The ZMBT mediated sulfuration of 2-pentene also follows the same mechanistic steps as shown in Fig. 7. However, in the case of 2-pentene, the allylic C–H bond is activated by the sulfur radical in **3**, which is followed by the subsequent C–S bond formation and S–S bond formation similar to pyrene (Fig. 7) to generate sulfurated 2-pentene-S–S-H (**9**) plus ZMBT (**1**). The sulfurated pyrene (**6**) and 2-pentene (**9**) reacts further to generate the desired crosslinked pyrene-2-pentene moiety, wherein H_2_S is eliminated as side product. Otherwise, two molecules of **6** or **9** reacts themselves to form the crosslinked pyrene or 2-pentene plus H_2_S.

**Figure 8:**
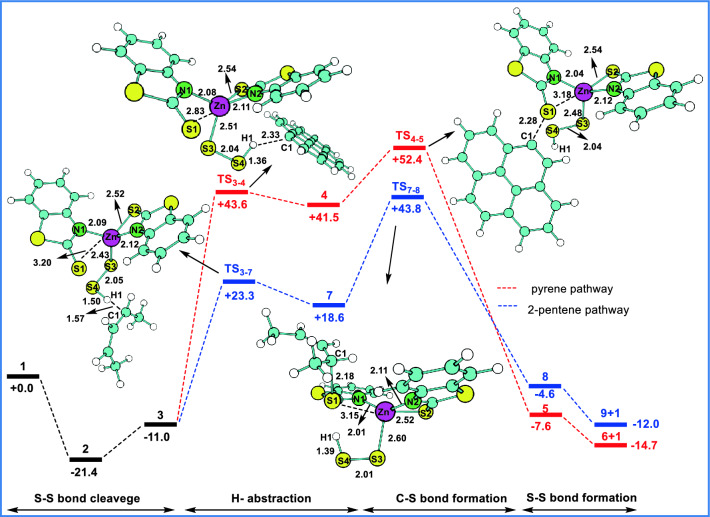
Computed free energy profile for the ZMBT mediated sulfuration of pyrene and 2-pentene. Optimized transition states are shown with bond distances (Å).

The computed free energy profiles for the sulfuration of pyrene and 2-pentene, along with the H-abstraction and C–S bond formation transition states are shown in Fig. 8. The relative free energy (ΔG) of all the intermediates is calculated with respect to the free energies of **1**, disulfur and pyrene or 2-penetene. In the first step, the disulfur atom (2S) is inserted into Zn-S1 bond of **1,** which results in the more stable species **2**, stable by − 21.4 kcal/mol compared to **1**. Due to sulfur insertion, **2** exhibits a more favorable 6-member ring structure compared to that of a 4-member ring structure in **1**. In the next step, the S1–S4 bond in **2** dissociates to form **3** (S1–S4 = 2.98 Å, ΔG = − 11.0 kcal/mol) wherein a lone pair electron is found on both S1 and S4 atoms (spin densities of S1 and S4 are 0.280 and 1.012, respectively) and generates a radical character to both S1 and S4 atoms. It is noted that, the intermediate **3** is found to be less stable than **2,** by + 10.4 kcal/mol and the lone pair electrons on sulfur atoms apparently activates the C–H bond of pyrene compared to that of **2**. In the next step, the C–H bond activation of pyrene is proposed via the H-abstraction by the sulfur radical (S4) in **3**. The H-abstraction step is associated with the transition state **TS**_**3-4**_ (Fig. 8, ΔG =  + 43.6 kcal/mol), wherein the C1–H1 and S4–H1 distances are found to be 2.33 Å and 1.36 Å, respectively. The TS_3-4_ generates **4** (ΔG =  + 41.5 kcal/mol) with the S4–H1 bond distance of 1.39 Å and leaving the lone pair electron on C1 and S3 (spin densities of C1 and S3 are 0.957 and 0.544, respectively). Further, **4** undergoes C–S bond formation through the **TS**_**4-5**_ (ΔG =  + 52.4 kcal/mol, C1–S1 = 2.28 Å), which is found to be higher than **TS**_**3-4**_ by + 8.8 kcal/mol. The intermediate **5** (ΔG = − 7.6 kcal/mol) is generated from **TS**_**4-5**_, wherein the C1–S1 bond is formed with the bond distance 1.8 Å. From **5**, the compound **6** is modelled by decreasing the S1–S4 (4.0 to 1.5 Å, using restricted optimization) bond distance in a step wise manner. While decreasing the S1-S4 bond distance of **5**, the Zn-sulfide moiety undergoes rearrangement in order to establish an interaction between the carbon atom of 2-mercaptobenzothiazole and the S3 atom. The unrestricted geometry optimization with the S1–S4 distance of 2.0 Å eventually leads to the formation of pyrene-S–S–H (**6**) and regenerates **1 (**ΔG = − 14.7 kcal/mol). In spite of many attempts, the location of **TS** of this step is not successful. However, the barrier height of ~  + 37.0 kcal/mol is estimated from the restricted calculations. The comparison of modelled transition states shows that **TS**_**3-4**_ is associated with higher activation barrier (ΔG^‡^ =  + 54.6 kcal/mol) than that of TS_4-5_ (ΔG^‡^ =  + 10.9 kcal/mol) and confirms the H-abstraction step as the rate determining step.

In the case of 2-pentene, the mechanistic steps involving intermediates **1–3** are as similar to pyrene. The allylic hydrogen is abstracted by the sulfur radical in **3** via **TS**_**3-7**_, (ΔG =  + 23.3 kcal/mol; C1-H1 = 1.57 Å, S4-H1 = 1.50 Å) and forms the intermediate **7** (ΔG =  + 18.6 kcal/mol; C1-H1 = 2.35 Å, S4-H1 = 1.36 Å). In **7**, the allylic C1-H1 bond (2.35 Å) is transformed to a S4-H1 (1.36 Å) bond creating the radical character on C1 (spin density of C1 = 0.194). In the next step, the C1-S1 bond formation takes place through **TS**_**7-8**_ (ΔG =  + 43.8 kcal/mol) with the C1-S1 bond distance of 2.18 Å and leads to the formation of **8** (ΔG = − 4.6 kcal/mol). Similar to **5**, the S1-S4 bond distance of **8** is systematically decreased, which results to the formation of 2-pentene-S–S-H (**9**) plus **1** (ΔG = − 12.0 kcal/mol). Here also, the attempts to locate the TS of this step is not fruitful. Overall, the computed free energy profile of 2-pentene shows that the transition state corresponds to the H-abstraction step, **TS**_**3-7**_ (ΔG^‡^ =  + 34.3 kcal/mol), is associated with the higher activation barrier compared to that of C-S bond formation transition state **TS**_**7-8**_ (ΔG^‡^ =  + 25.2 kcal/mol)_,_ by + 9.1 kcal/mol. This indicates the H-abstraction step is the rate determining step for the sulfuration of 2-pentene, similar to the sulfuration of pyrene.

The comparison of the energetics of the rate determining transition states of pyrene (**TS**_**3-4**_) and 2-pentene (**TS**_**3-7**_) indicates that sulfuration of 2-pentene is kinetically more feasible than the sulfuration of pyrene by + 20.3 kcal/mol. This is likely due the more reactive allylic C-H bond of 2-pentene, which comprises early transition state (**TS**_**3-7**,_ C1–H1 = 1.57 Å) for the H-abstraction step compared to that of **TS**_**3-4**_ (C1–H1 = 2.33 Å) in pyrene. However, it is found that the sulfurated pyrene-S–S–H (**6**) is found to be thermodynamically slightly more stable than the sulfurated 2-pentene-S–S–H (**9**) by + 1.3 kcal/mol.

**Figure 9:**
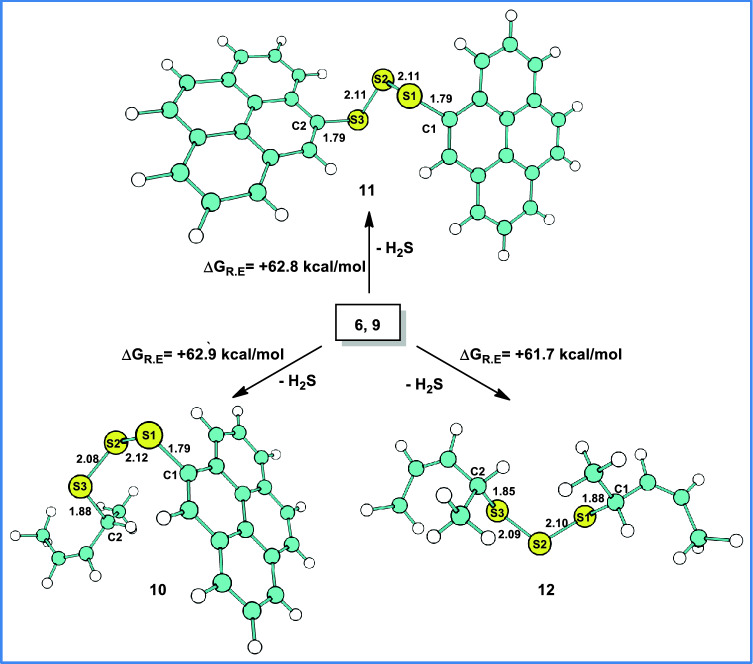
Optimized crosslinked reaction product geometries from sulfurated pyrene and/or 2-pentene, **10**, **11** and **12**, respectively. Optimized bond lengths are shown in Å.

**Figure 10:**
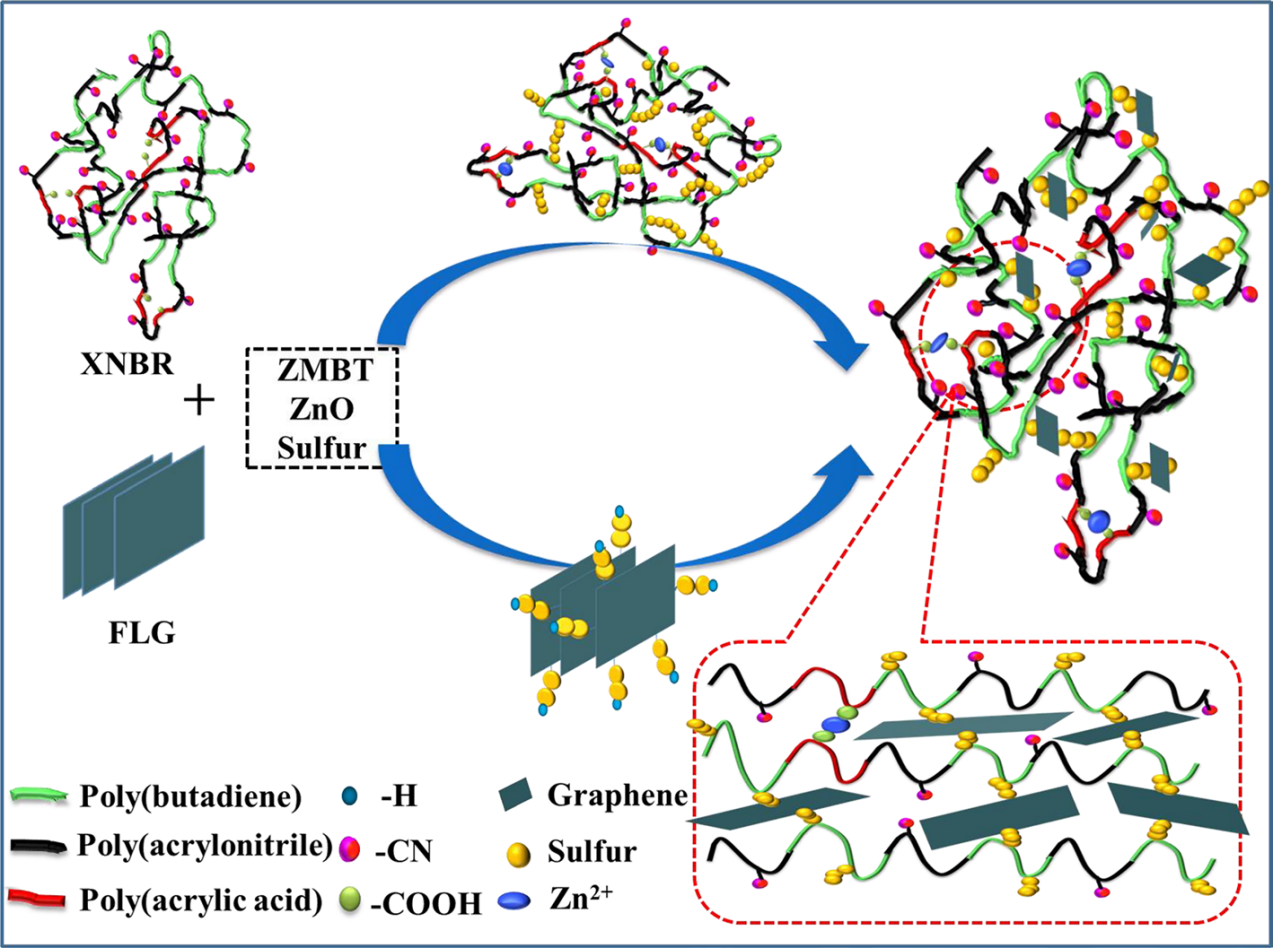
Illustration of formation of the covalently edge linked XNBR-FLG nanocomposites via sulfur.

Crosslinked products from sulfurated pyrene (**6)** and/or 2-pentene (**9)** are considered for further reactions to form **10, 11** and **12** along with H_2_S (Fig. 9) elimination. The formation energies of **10, 11** and **12** are found to be similar, namely, + 62.9, + 62.8 and + 61.7 kcal/mol, respectively. This implies that the formation of these products is endothermic in nature and requires higher temperatures. Experimentally, the ZMBT assisted crosslinking of XNBR with sulfur was carried out at 70 °C (here modeled as **12**). Consequently, the predicted energies suggest that **10** and **11** could also be formed at the same temperature and supports the hypothesis of the formation of covalently edge linked FLG-XNBR nanocomposites through sulfur (Fig. 10).

## Conclusions

This study indicates that elemental sulfur can react with few layers graphene to form edge sulfurated graphene layers. XPS provides evidence for edge C-S bonds in graphene, which can chemically react with XNBR during the curing process. This may lead to the formation of chemical linkages between FLG and XNBR. Such strong covalent interaction between the FLG and the rubber could result in better dispersion of the FLG in the rubber matrix resulting in improved mechanical property of the nanocomposites. Additionally, higher equilibrium swelling and lower crosslinking density were observed for the nanocomposites compared to XNBR. The analysis of results using Lorentz-Park and Cunneen-Russell models suggest a strong interaction between FLG and XNBR. DFT study utilizing simple models viz*.* pyrene and 2-pentene provides further mechanistic insights. The computed energetics indicate that the H-abstraction step is the rate determining step for the sulfuration of pyrene and 2-pentene. We propose that chemical bond formation between FLG edges with rubbers via the formation of S–S bonds leads to superior dispersion and improved physical properties of FLG-rubber thin film nanocomposites.

## Experimental

### Analysis

High resolution transmission electron microscope (JEOL, JEM 2100F) equipped with an EDS was employed to analyze the aqueous dispersions of the sulfurated FLG, which was drop cast on to TEM grid. X-Ray photoelectron analysis of the samples were performed using XPS VG Multilab ESCA XPS (Model: 220i) with Mg/Al Kα radiation. UV/Vis spectrometer (Shimadzu UV-1700) was used for estimation of ZMBT. The Raman spectra were recorded using a Confocal Raman Microscope (alpha 300RA, Witec, Germany) with 532 nm laser.

## Computational study

All geometries are fully optimized by the B3LYP-DFT method using the Gaussian 16 program^49^. The calculations were carried out with a mixed basis set (B1) of LanL2DZ for the Zn center, which has a relativistic effective core potential with a valence basis set and 6-31G** for the remaining atoms^50^. All the intermediates were confirmed by the frequency calculations as minima on the potential energy surface. The transition states were characterized by a single negative frequency. It is further verified by animating the frequency that correspond to the C-H bond elongation or C–S bond formation, as concern to this study. For selected transition states, intrinsic reaction coordinate (IRC) calculations were performed to confirm that the transition state is indeed connected to the corresponding reactant and the product in the potential energy surface. Further, to get the reliable energetics, single-point calculations were performed on the B3LYP/B1-optimized geometries using LanL2DZ for the Zn center and 6–311 +  + G** for the remaining atoms (basis set B2). The quoted energies are those calculated at the B3LYP/B2//B3LYP/B1 level, including the free energy corrections obtained from the B3LYP/B1-optimized geometries.

### Materials and methods

Carboxylated nitrile butadiene rubber (XBN 500: % of butadiene, acrylonitrile and methacrylic acid are 66, 28 and 6, respectively, and having ~ 4.3 wt.% nonvolatile matter) was bought from Apcotex Industries Limited, Taloja, India. Graphite and melamine were procured from Sigma Aldrich, Switzerland and USA, correspondingly. Toluene and chloroform were supplied by Spectrochem, India and used as such. Sulfur (crosslinking agent) was purchased from Associate Chemicals, Kochi, India. Accelerator ZMBT was procured from Merck Chemicals, India. Zinc oxide (ZnO) is employed as an activator for crosslinking and was bought from Nice Chemicals, India. Sodium poly(naphthalene sulfonate) (an anionic surfactant) was procured from Vanderbilt, USA.

### Compounding of XNBR latex, preparation of FLG-aqueous dispersion and mixing with compounded XNBR latex

Compounding ingredients *(*sulfur, 9.6 g, ZnO, 19.3 g, ZMBT, 9.6 g and 200 mL water) were transferred to a sand grinder (Diamill, Abigail Enterprises, model S0.3) and subjected to continuous grinding for 1 h. To this, 1 L of XNBR latex was added. The pH of the resulting dispersion was adjusted to 8.5 using 5% KOH and stirred in a tank for 2 days at 25 °C. The produced FLG (24.7 g) was dispersed in water using the probe sonicator (SONICS, 750 W for 2 min at 25% amplitude). The aqueous-FLG dispersion was mixed with compounded XNBR using Microfluidizer (M700 – Microfluidics) at 1.38 × 10^8^ Pa. 50 mL of microfludized XNBR-FLG nanocomposite latex was poured into glass plate (16 × 16 × 0.5 cm) and allowed to dry at room temperature and vulcanized at 80 ºC for 45 min. Vulcanized nanocomposite sheets were taken for further analysis.

### Synthesis of edge sulfurated few layers graphene

20 mg of FLG [contains 1:3:0.13 mixture of FLG, melamine and poly(naphthalene sulfonate)] was dispersed in 50 mL of distilled water. The mixture was sonicated for 15 min (SONICS, 750 W, at 25% amplitude) to get FLG-aqueous dispersion. Sulfur, ZnO and ZMBT (5, 10 and 5 mg, respectively) was subjected to grinding in a planetary ball mill (A RetschPM 400 with 4 grinding bowl fasteners) at 100 rpm for 2 h (successive grinding with 15 min grinding and 15 min pause) and the resulting ground product was mixed with 50 mL of FLG-aqueous dispersion. The dispersion was kept in Radleys Carousel 12 plus Reaction Station for 24 h at 70/100 °C. A re-circulating chiller (F-105 Buchi, Switzerland) was also connected to the reactor to condense the water evaporating from the reaction medium. Two control experiments were also performed i.e. only with FLG and sulfur at 70 and 100 °C. The resulting solid product mixture was purified by repeated hot water washing followed by Soxhlet extraction (24 h) using CHCl_3_.

## Supplementary Information

Supplementary Information.
