# Supplementary material for: In-situ interfacial compatibilization via edge-sulfurated few layer graphene during the formation of crosslinked graphene-rubber nanocomposites
Source: Sci Rep. 2022 Mar 7;12:4013. doi: 10.1038/s41598-022-08071-w (PMC8901685; doi:10.1038/s41598-022-08071-w)
Supplement: Supplementary file 1 — Supplementary Information. [file 41598_2022_8071_MOESM1_ESM.docx]

**SUPPORTING INFORMATION**

**In-situ interfacial compatibilization via edge-sulfurated few layer graphene during the formation of crosslinked graphene-rubber nanocomposites**

Sangeeth Krishnan^1^_,_ Maya M.G^1^, Akhil Das^1^, Suja Bhargavan^1^, Krithika Ganesan^2^, Swaminathan Sivaram^3^, Prabha Vadivelu^2*^and Lakshminarayanan Ragupathy^1*^

^1^Corporate R&D Center, HLL Lifecare Limited, Akkulam, Sreekariam (P.O), Thiruvananthapuram 695017, India

^2^Deapartment of Chemistry, Central University of Tamil Nadu, Neelakudi, Thiruvarur 610005, India

^3^Indian Institute of Science Education and Research, Dr. Homi Bhabha Road, Pune 411008, India

^2^[^*^prabhav@cutn.ac.in](mailto:*prabhav@cutn.ac.in) and ^1*^laks@lifecarehll.com

**CONTENT**

| Table S1 | The literature comparison of different XNBR/graphene nanocomposites | S2 |
| --- | --- | --- |
| Section 1 | Swelling studies on XNBR-FLG nanocomposites | S3 |
| Table S2 | Swelling properties of XNBR and XNBR-FLG nanocomposite thin films by micro fluidization process | S4 |
| Section 2 | Estimation of remaining ZMBT after purification of sulfurated FLG | S4 |
| Figure S1 | The UV absorption spectrum of remaining ZMBT after purification of sulfurated FLG | S5 |
| Table S3 | Collective standardization data of ZMBT stock solution in chloroform solvent | S6 |
| Figure S2 | Collective standard curve of ZMBT in chloroform solvent. | S6 |
| Table S4 | Combined UV analysis data of sulfurated FLG | S7 |
| Section 3 | DFT investigation on sulfuration of pyrene and 2-pentene coordinates of optimized geometries | S9 |

**Table S1**: The literature comparison of different XNBR/graphene nanocomposites.

| **Composites** | **Method of mixing of graphene with rubber** | **Lab/ Industrial scale** | **Tensile properties [Tensile strength/modulus (MPa) and elongation at break (%)]** | **Interfacial interaction between graphene and XNBR** | **Ref.** |
| --- | --- | --- | --- | --- | --- |
| f GO-XNBR | Solution mixing: Hexadecyl amine functionalized GO is mixed with XNBR in THF | Lab (5 g) | Control: 2.9/1.57 and 227 | Non-Covalent interaction between XNBR and fGO | ^1^ |
|  |  |  | fGO/XNBR: 4.6/1.35 and 365 |  |  |
| f GO-XNBR | Latex mixing: XNBR by sonication and latex coagulation with CaCl_2_ | Lab (20 g) | Control: 16.5/5 and 350 | H-bonding and π-π interaction between Sodium Humate and graphene layer | ^2^ |
|  |  |  | fGO/XNBR: 36/5 and 450 |  |  |
| EG-XNBR | Latex mixing: *EG* was added to XNBR latex by ultrasonic irradiation followed by latex coagulation with CaCl_2_ | Lab (not provided) | Control: 7.4/1.3 and 590 | H-bonding (through -COOH group in EG) | ^3^ |
|  |  |  | EG/XNBR: 12.2/2.4 and 590 |  |  |
| GO-XNBR | *GO* was mixed with XNBR in laboratory two-roll mill. | Lab (not provided) | Control: 3.6/0.8 and 504 | H- bonding (through -COOH, -OH groups in GO) | ^4,5^ |
|  |  |  | GO/XNBR: 16/2.3 and 373 |  |  |
| FLG-XNBR | Latex mixing: FLG was mixed with XNBR latex by PS, HPH and Industrial MF | Lab  (PS &HPH 1-2 L)  Industrial (MF-20 L) | Control: 6.7/0.01 and 688 | Covalent (sulfurated FLG and XNBR) and Non-covalent interactions | Present study |
|  |  |  | FLG/XNBR: 13.4/0.02 and 588 |  |  |

**Section 1: Swelling studies on XNBR-FLG nanocomposites**

The solubility parameter of XNBR and XNBR graphene nanocomposites was calculated by group contribution method to select the most compatible solvents to conduct the swelling studies.^6^ On this basis two solvents namely, toluene and chloroform were used for transient swelling experiments and the interaction parameter χ between nanocomposites and solvents was calculated using equation (1)

$$= + V_{S}\left( \frac{{({}_{P}-{}_{S})}^{2}}{RT} \right) (1)$$

here, β is the lattice constant with value 0.34, $V_{s}$ is the molar volume of solvent, *R* is the universal gas constant, *T* is the temperature in (K), ${}_{P}$ and ${}_{S}$ are the solubility parameter of XNBR nanocomposites and solvent, respectively. The samples of dimension 1 cm $\times$ 2 cm $\times0.6 \mathrm{mm}$ was used for swelling experiments at room temperature. Before conducting the experiments, the thickness (h) and initial weight (M_0_) of the samples were noted. The samples were immersed in solvents and the weight increase of the films at different time intervals (M_t_) were measured in an analytical balance (Shimadzu, model auw220) by taking out the swollen samples and wiping out the excess solvent adhered on the film surface using cotton. The samples were weighed quickly and reimmersed in the solvent to minimize the error induced by evaporation of the solvents. This procedure is continued until we obtain a constant weight of the swollen films and the weight of swollen films at the highest duration of solvent interaction (24 h) was taken to be the “equilibrium swollen condition” and the weight of swollen film at the equilibrium swelling is notated as$M_{\infty}$. The equilibrium swelling ratio ‘S’ is calculated by equation (2)

$$S= \frac{M_{\infty}-M_{0}}{M_{0}} (2)$$

The solvent molecules diffuse in to the polymer matrix until the elastic retraction of the polymer network balances the osmotic pressure driving the solvent in to the swollen polymer. The elastic retraction depends on the crosslinking density ($\vartheta$) which is calculated using Flory-Rehner equation (3).

$$\vartheta= \frac{\ln\left( 1- \right)++{}^{2}}{2\times({}_{p}\times V_{S}\times{}^{\frac{1}{3}})} (3)$$

**Table S2:** Swelling properties of XNBR and XNBR-FLG nanocomposite thin films by microfluidization process

| **Film** | **Equilibrium swelling** | | **Cross Link Density (mol/cm^3^)**  $\boldsymbol{\times10}$**^4^** | |
| --- | --- | --- | --- | --- |
|  | **Toluene** | **CHCl_3_** | **Toluene** | **CHCl_3_** |
| XNBR (control) | 2.5 | 10.0 | 2.4 | 1.0 |
| XNBR- FLG 1 | 3.9 | 23.4 | 1.0 | 0.2 |
| XNBR- FLG 3 | 3.7 | 22.0 | 1.1 | 0.3 |
| XNBR- FLG 5 | 4.8 | 21.8 | 0.7 | 0.2 |
| XNBR- FLG -10 | 4.4 | 27.9 | 0.8 | 0.2 |

**Section 2: Estimation of remaining ZMBT after purification of sulfurated FLG**

In the case of FLG and XNBR, it is expected to have non-covalent interactions *viz.* van der Waals, π-π and hydrophobic interactions. These interactions however, are weak and may not able to increase the tensile properties up to 100% with small amount of the filler. In general, the covalent attachment between the filler and polymer can be very efficient in strengthening the material and suggests that there may be a covalent interaction between and FLG and XNBR. Thus, we hypothesized that FLG could be chemically linked with XNBR through its edges via sulfur. To prove our theory, a reaction between compounding ingredients *viz.* sulfur, ZnO and Zinc-2-mercaptobenzothiozole (ZMBT) and FLG (1:3:0.13 ratio of FLG, melamine and surfactant) in water at 70 °C for 24 h. Then the reaction mixture was washed with hot water (to remove melamine) followed by soxhlet extraction in CHCl_3_ (to remove the un-reacted sulfur and ZMBT) and performed XPS analysis. From XPS, we observed S 2p peak is due to sulfurated FLG and not because of the remaining ZMBT after the soxhlet extraction. Hence UV analysis of the same sample was performed in accordance with CHCl_3_ to confirm this observation.

Based on the solubility of analyte, different solvents were used for UV analysis. Here, we tried chloroform for ZMBT. The absorption spectrum of ZMBT (1 ppm) from 200-800 nm was obtained from spectrometer (Figure S2). As we know that the ultraviolet region (about 400-190) is particularly important for the quantitative and qualitative determination of different organic compounds, especially those with a high degree of conjugation. The basic principle of qualitative absorption spectroscopy lies in comparing the extend of absorption of a sample solution with that of a set of standards under radiation of a selected wavelength through the application of Beer-Lambert law. Here the spectrum shows a broad absorption at 323nm wavelength.


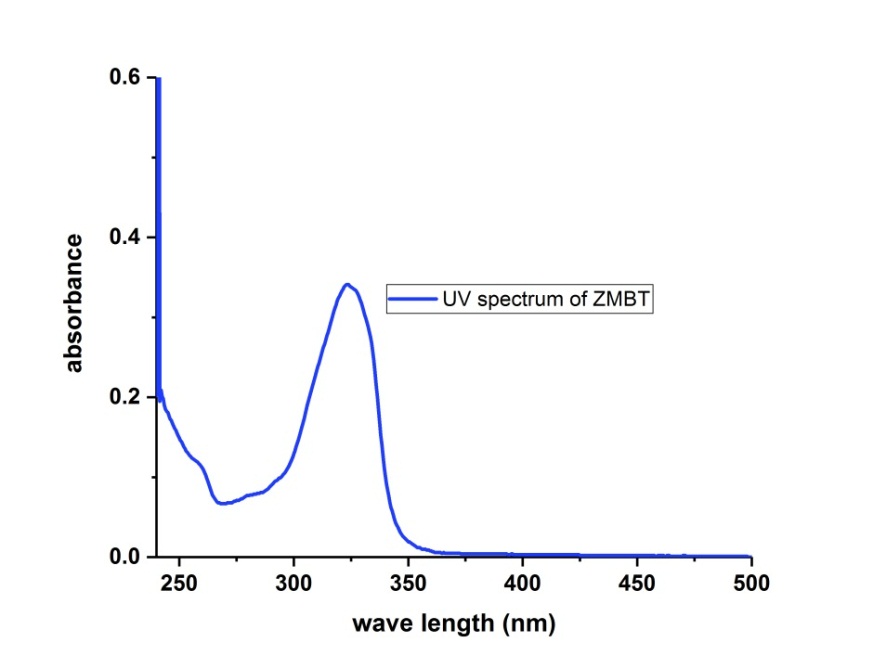


**Figure S1:** The UV absorption spectrum of remaining ZMBT after purification of sulfurated FLG.

To make a standard curve of for ZMBT, we prepared a stock solution of ZMBT by accurately measuring 1mg of ZMBT using microbalance and made up to 1L. From that stock solution, we prepared serial dilution containing concentration 0.5, 1.0, 2.0, 3.0 and 5.0. The standard curve was plotted with absorbance against concentration. Three standard curves were plotted and the combined data is shown in table S2. Figure S3 shows the combined standard curve of ZMBT in chloroform solvent.

**Table S3**: Collective standardization data of ZMBT stock solution in chloroform solvent

|  | **Sample ID** | **Type** | **Concentration (ppm)** | **Wavelength 323 nm** | **Weight factor** |
| --- | --- | --- | --- | --- | --- |
| 1 | Std 1 | Standard | 0.500 | 0.063 ± 0.003 | 1.00 |
| 2 | Std 2 | Standard | 1.000 | 0.110 ± 0.002 | 1.00 |
| 3 | Std 3 | Standard | 2.000 | 0.221 ± 0.006 | 1.00 |
| 4 | Std 4 | Standard | 3.000 | 0.335 ± 0.006 | 1.00 |
| 5 | Std 5 | Standard | 5.000 | 0.556 ± 0.003 | 1.00 |

The sample mixture contains 40% few layers graphene and 60% compounded chemicals (Sulfur, ZMBT, ZnO). The compounded chemicals are taken in the ratio 1:2:1 (Sulfur: ZnO: ZMBT). So, once we are taking 10 mg of sample mix, it will contain about 1.5 mg of ZMBT. Therefore; for 1 mg it will be 0.15 mg ZMBT.


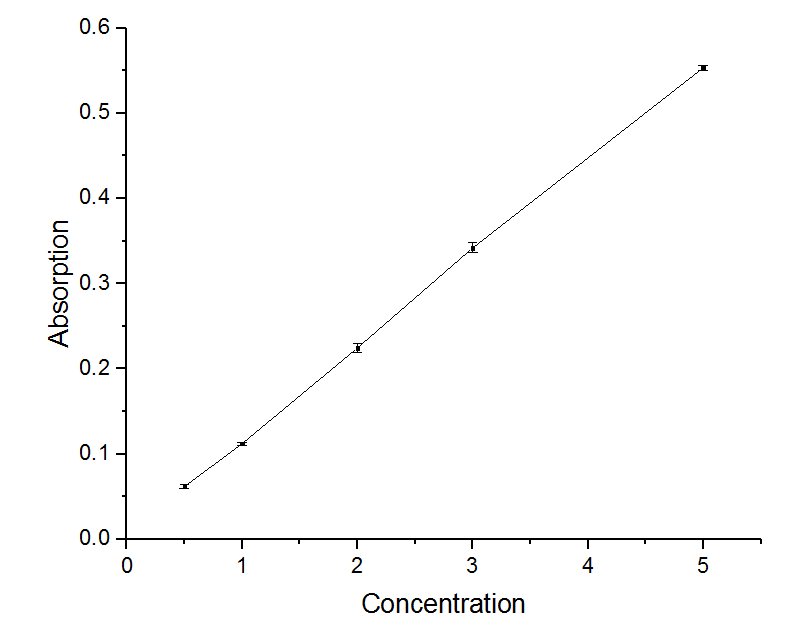
 **Figure S2:** Collective standard curve of ZMBT in chloroform.

**Table S4**: Combined UV analysis data of sulfurated FLG.

| **S. No.** | **Sample** |  | **Concentration (ppm)** |
| --- | --- | --- | --- |
| 1 | Sulfurated FLG | Standard Curve 1 | 0.7028 |
| 2 |  | Standard Curve 2 | 0.6373 |
| 3 |  | Standard Curve 3 | 0.7260 |
|  | | | - 1. ±0.046 |

**Table S5: Elemental composition of FLG and Sulfurated FLG from XPS analysis.**

| Sl. No. | Sample ID | XPS | | | | |
| --- | --- | --- | --- | --- | --- | --- |
|  |  | **S2P (%)** | **C1s (%)** | **N1s (%)** | **O1s (%)** | **Zn2p (%)** |
| 1 | FLG | - | 98 | 2 | - | - |
| 2 | Sulfurated FLG | 0.73 | 88.84 | 4.3 | 5.33 | 0.8 |

**Section 3: DFT investigation on sulfuration of pyrene and 2-pentene: Coordinates of optimized geometries**

ZMBT (1)

6 -4.799981000 2.398748000 -1.412168000

6 -5.027033000 1.599990000 -0.291889000

6 -3.581784000 2.335258000 -2.106306000

1 -3.428825000 2.965451000 -2.976953000

6 -4.010608000 0.738203000 0.119292000

6 -2.566061000 1.476170000 -1.697785000

1 -1.623375000 1.420525000 -2.232729000

6 -2.780408000 0.669564000 -0.573442000

1 -5.577612000 3.076539000 -1.750087000

6 -2.356304000 -0.871611000 1.037636000

6 2.565939000 1.476478000 1.697523000

6 2.780353000 0.669603000 0.573356000

6 3.581546000 2.335774000 2.105815000

1 3.428568000 2.966163000 2.976318000

6 4.010559000 0.738291000 -0.119344000

6 4.799749000 2.399277000 1.411654000

1 5.577258000 3.077254000 1.749480000

6 5.026902000 1.600309000 0.291578000

1 5.969616000 1.648767000 -0.243850000

1 1.623244000 1.420842000 2.232457000

16 3.990325000 -0.387705000 -1.477514000

7 1.881917000 -0.238671000 0.030327000

6 2.356516000 -0.871917000 -1.037377000

7 -1.881839000 -0.238573000 -0.030230000

16 -3.990213000 -0.387376000 1.477805000

1 -5.969779000 1.648474000 0.243481000

16 -1.385273000 -2.048897000 1.853475000

16 1.385290000 -2.049295000 -1.853051000

30 0.000066000 -1.235858000 -0.000050000

ZMBT-2S (2)

6 4.636663000 0.124314000 2.817275000

6 4.879039000 0.647556000 1.547542000

6 3.419834000 -0.508956000 3.114518000

1 3.255649000 -0.911700000 4.109269000

6 3.879432000 0.525122000 0.582720000

6 2.420571000 -0.631812000 2.153804000

1 1.479117000 -1.124934000 2.374160000

6 2.650398000 -0.109038000 0.875147000

1 5.401616000 0.207393000 3.583029000

6 2.252705000 0.420904000 -1.294311000

6 -0.927385000 2.480552000 -0.567536000

6 -1.843868000 1.569916000 -0.023364000

6 -1.194688000 3.840380000 -0.458752000

1 -0.492366000 4.551695000 -0.881333000

6 -3.007708000 2.051423000 0.612206000

6 -2.354955000 4.307521000 0.179154000

1 -2.538527000 5.374972000 0.250332000

6 -3.277625000 3.416919000 0.719663000

1 -4.177223000 3.772543000 1.210830000

1 -0.042915000 2.129177000 -1.086352000

16 -3.955433000 0.702491000 1.204746000

7 -1.738075000 0.169294000 -0.060120000

6 -2.759102000 -0.404084000 0.536849000

7 1.766654000 -0.142530000 -0.194526000

16 3.880265000 1.069464000 -1.095061000

1 5.821343000 1.134499000 1.317813000

16 1.307795000 0.470770000 -2.740591000

16 -3.134158000 -2.121488000 0.704593000

30 -0.052642000 -0.826684000 -1.090636000

16 -1.246428000 -3.074161000 0.674375000

16 -0.600000000 -3.103624000 -1.319666000

ZMBT-2S_rad_ (3)

6 5.363710000 -0.216655000 2.270946000

6 5.311244000 0.771090000 1.287950000

6 4.277596000 -1.080233000 2.481950000

1 4.341063000 -1.840327000 3.254556000

6 4.151353000 0.875593000 0.520626000

6 3.118863000 -0.978706000 1.717795000

1 2.272430000 -1.638737000 1.877109000

6 3.055114000 0.007865000 0.726236000

1 6.256998000 -0.315904000 2.879743000

6 2.194788000 1.256721000 -0.968582000

6 -2.696439000 2.325646000 -0.362673000

6 -2.703153000 1.111585000 0.340486000

6 -3.775031000 3.186809000 -0.204968000

1 -3.786379000 4.130396000 -0.741280000

6 -3.785450000 0.788571000 1.192665000

6 -4.849621000 2.858563000 0.640446000

1 -5.679838000 3.549973000 0.745650000

6 -4.865074000 1.659940000 1.349508000

1 -5.693634000 1.410989000 2.004374000

1 -1.856137000 2.575822000 -1.002616000

16 -3.501326000 -0.774539000 1.941576000

7 -1.710604000 0.143912000 0.304404000

6 -1.964140000 -0.907056000 1.084648000

7 1.984228000 0.249965000 -0.124505000

16 3.777687000 2.006771000 -0.780764000

1 6.150779000 1.439499000 1.125990000

16 0.979753000 1.710453000 -2.104814000

16 -0.891877000 -2.235864000 1.202728000

30 0.036627000 -0.220448000 -0.774594000

16 -1.286097000 -3.438494000 -1.500228000

16 -0.454516000 -1.981691000 -2.547699000

TS_3-4_

6 -7.140968000 -2.721780000 -1.920499000

6 -7.459105000 -1.949990000 -0.804319000

6 -5.816865000 -2.814771000 -2.380115000

1 -5.595494000 -3.419994000 -3.253717000

6 -6.425554000 -1.275896000 -0.152696000

6 -4.783935000 -2.142013000 -1.736267000

1 -3.759002000 -2.201482000 -2.089209000

6 -5.091211000 -1.364776000 -0.611311000

1 -7.929343000 -3.257347000 -2.440191000

6 -4.752466000 0.033144000 1.154477000

6 -1.478726000 3.718902000 -0.030996000

6 -0.897566000 2.697896000 -0.793427000

6 -1.142650000 5.037908000 -0.321470000

1 -1.586114000 5.840688000 0.259601000

6 0.005456000 3.015839000 -1.833204000

6 -0.242550000 5.347147000 -1.353414000

1 0.003486000 6.384020000 -1.560577000

6 0.340372000 4.339016000 -2.120623000

1 1.034689000 4.579133000 -2.919409000

1 -2.178466000 3.468327000 0.759930000

16 0.558385000 1.533364000 -2.612619000

7 -1.115521000 1.334839000 -0.640721000

6 -0.437210000 0.590646000 -1.508602000

7 -4.190966000 -0.621554000 0.138212000

16 -6.495262000 -0.237722000 1.266953000

1 -8.482664000 -1.878554000 -0.450965000

16 -3.835312000 1.011068000 2.221076000

16 -0.588024000 -1.128509000 -1.475066000

30 -2.152205000 -0.237192000 0.318460000

16 0.431447000 -0.961528000 2.863244000

16 -1.559275000 -1.297347000 2.522686000

1 0.889192000 -0.606739000 1.626155000

6 4.350489000 -2.514920000 -1.104108000

6 5.731448000 -2.227890000 -0.823518000

6 3.348297000 -1.694640000 -0.683104000

6 6.052507000 -1.045544000 -0.088421000

6 3.644786000 -0.502264000 0.063764000

6 5.013635000 -0.176687000 0.357553000

1 4.117069000 -3.412924000 -1.670038000

6 6.774144000 -3.066793000 -1.249327000

6 7.416320000 -0.732147000 0.200393000

6 5.329492000 1.011626000 1.088726000

6 8.423161000 -1.602868000 -0.246805000

6 8.102315000 -2.755522000 -0.962324000

1 6.533643000 -3.966855000 -1.808783000

6 7.705656000 0.466983000 0.938992000

1 9.461540000 -1.367574000 -0.028823000

1 8.894860000 -3.417032000 -1.299990000

6 2.704005000 0.395557000 0.546325000

6 4.295066000 1.861506000 1.516245000

6 2.954482000 1.558146000 1.239968000

1 2.309394000 -1.914610000 -0.908794000

6 6.712791000 1.297674000 1.360999000

1 8.744809000 0.700100000 1.155893000

1 4.540052000 2.765014000 2.069062000

1 2.157103000 2.218383000 1.568430000

1 6.951761000 2.200081000 1.917458000

4

6 -5.709512000 -3.883281000 -2.844996000

6 -6.501091000 -3.347567000 -1.829332000

6 -4.361957000 -3.513903000 -2.977135000

1 -3.766232000 -3.941808000 -3.777668000

6 -5.916752000 -2.437973000 -0.948320000

6 -3.775488000 -2.606200000 -2.099978000

1 -2.736333000 -2.308867000 -2.198414000

6 -4.559652000 -2.064095000 -1.074145000

1 -6.144353000 -4.593502000 -3.541462000

6 -5.098869000 -0.795276000 0.736967000

6 -3.060712000 3.902051000 0.558428000

6 -2.062057000 3.204495000 -0.132731000

6 -3.129086000 5.283302000 0.407489000

1 -3.898000000 5.839096000 0.935050000

6 -1.156021000 3.899553000 -0.964658000

6 -2.222692000 5.969393000 -0.416967000

1 -2.297159000 7.047752000 -0.517353000

6 -1.227639000 5.285060000 -1.113102000

1 -0.530109000 5.816248000 -1.752582000

1 -3.762549000 3.360804000 1.184864000

16 -0.035913000 2.756183000 -1.702855000

7 -1.848190000 1.831570000 -0.098429000

6 -0.839583000 1.434158000 -0.861323000

7 -4.140563000 -1.150178000 -0.116590000

16 -6.641151000 -1.591096000 0.419272000

1 -7.543715000 -3.631747000 -1.728834000

16 -4.776892000 0.330559000 1.996504000

16 -0.426867000 -0.239229000 -0.984288000

30 -2.580643000 0.014031000 0.677496000

16 0.642306000 -0.879443000 2.339425000

16 -1.337006000 -0.871791000 2.774640000

1 0.532755000 -0.583207000 0.981334000

6 4.423472000 -0.311237000 -1.902836000

6 5.860439000 -0.346773000 -1.876667000

6 3.682074000 -0.465684000 -0.770912000

6 6.519878000 -0.550076000 -0.625655000

6 4.320316000 -0.671902000 0.500895000

6 5.756341000 -0.713679000 0.567542000

1 3.929414000 -0.156878000 -2.858414000

6 6.636408000 -0.188820000 -3.036636000

6 7.947187000 -0.590083000 -0.569634000

6 6.412811000 -0.918148000 1.821856000

6 8.678965000 -0.426865000 -1.757121000

6 8.028367000 -0.228848000 -2.974076000

1 6.137365000 -0.034349000 -3.989549000

6 8.580932000 -0.796717000 0.703979000

1 9.764626000 -0.456878000 -1.717741000

1 8.611583000 -0.104806000 -3.881931000

6 3.666952000 -0.841061000 1.712151000

6 5.647576000 -1.078578000 2.989817000

6 4.247432000 -1.041002000 2.943009000

1 2.597585000 -0.434857000 -0.813986000

6 7.850503000 -0.952983000 1.842510000

1 9.666740000 -0.826265000 0.740470000

1 6.151227000 -1.233988000 3.940734000

1 3.657146000 -1.165428000 3.845726000

1 8.347658000 -1.108457000 2.796323000

TS_4-5_

6 -4.062094000 -4.365658000 -2.782730000

6 -5.011046000 -3.977478000 -1.837412000

6 -2.803512000 -3.746614000 -2.833157000

1 -2.082571000 -4.060995000 -3.581743000

6 -4.673914000 -2.961638000 -0.943322000

6 -2.463761000 -2.732423000 -1.942644000

1 -1.497884000 -2.239891000 -1.981103000

6 -3.406512000 -2.336484000 -0.985405000

1 -4.303949000 -5.154212000 -3.488685000

6 -4.305988000 -1.172318000 0.746742000

6 -2.822254000 3.636607000 0.914794000

6 -1.884614000 3.134793000 -0.000861000

6 -3.185595000 4.977879000 0.838831000

1 -3.912356000 5.370992000 1.543224000

6 -1.339624000 3.996674000 -0.981648000

6 -2.633425000 5.826088000 -0.132313000

1 -2.932259000 6.868891000 -0.172834000

6 -1.705039000 5.339728000 -1.053604000

1 -1.280461000 5.991430000 -1.810727000

1 -3.262769000 2.978373000 1.656536000

16 -0.223488000 3.098938000 -2.005466000

7 -1.411035000 1.832788000 -0.055470000

6 -0.532843000 1.619517000 -1.064278000

7 -3.236985000 -1.346878000 -0.025957000

16 -5.643575000 -2.250846000 0.346491000

1 -5.985859000 -4.453251000 -1.800377000

16 -4.300150000 0.009080000 1.998714000

16 0.257898000 0.141239000 -1.336907000

30 -2.012885000 0.122273000 0.897211000

16 1.148486000 0.329856000 2.499045000

16 -0.574727000 -0.755534000 2.726802000

1 1.863374000 -0.240128000 3.498831000

6 4.895796000 -1.288809000 0.047446000

6 3.733458000 -2.107241000 0.203384000

6 6.187697000 -1.840726000 0.291957000

6 3.887579000 -3.450137000 0.597520000

6 6.291413000 -3.190162000 0.683565000

6 5.154502000 -3.981964000 0.833304000

1 3.003146000 -4.070532000 0.714569000

1 7.276137000 -3.610926000 0.868859000

1 5.256321000 -5.020112000 1.135197000

6 4.760836000 0.074693000 -0.349491000

6 3.461452000 0.631308000 -0.603020000

6 5.925626000 0.884899000 -0.503450000

6 3.370063000 1.975525000 -1.003969000

6 5.776481000 2.227122000 -0.902601000

6 4.515526000 2.760370000 -1.149260000

1 2.392928000 2.401177000 -1.201427000

1 6.663564000 2.844157000 -1.019581000

1 4.418764000 3.796440000 -1.459639000

6 2.319016000 -0.213555000 -0.425556000

6 2.447080000 -1.522057000 -0.036741000

6 7.339789000 -1.000144000 0.131183000

6 7.213183000 0.303372000 -0.249373000

1 8.094283000 0.928415000 -0.368822000

1 8.322016000 -1.425491000 0.319003000

1 1.563531000 -2.144624000 0.096163000

5

6 -4.012127000 -5.194219000 -0.532807000

6 -5.061938000 -4.276459000 -0.554180000

6 -2.711230000 -4.787219000 -0.200394000

1 -1.909434000 -5.519220000 -0.183213000

6 -4.783672000 -2.946367000 -0.240790000

6 -2.431292000 -3.460054000 0.112060000

1 -1.428747000 -3.142253000 0.376516000

6 -3.474211000 -2.525150000 0.088074000

1 -4.207382000 -6.234910000 -0.772373000

6 -4.533623000 -0.530991000 0.296520000

6 -2.303723000 3.451595000 -1.236558000

6 -1.216209000 2.580868000 -1.394697000

6 -2.270496000 4.677464000 -1.889245000

1 -3.106417000 5.360546000 -1.778102000

6 -0.125956000 2.955952000 -2.210235000

6 -1.175932000 5.047116000 -2.689617000

1 -1.176418000 6.011686000 -3.187711000

6 -0.090485000 4.192710000 -2.858532000

1 0.752744000 4.476224000 -3.479539000

1 -3.150962000 3.155420000 -0.629008000

16 1.062781000 1.667181000 -2.238593000

7 -1.072242000 1.329033000 -0.788965000

6 0.045676000 0.742749000 -1.142095000

7 -3.370903000 -1.169943000 0.373464000

16 -5.883395000 -1.571757000 -0.164848000

1 -6.069899000 -4.591286000 -0.805039000

16 -4.644819000 1.151335000 0.649988000

16 0.430841000 -0.901292000 -0.629272000

30 -2.222029000 0.567631000 0.950790000

16 -0.965409000 2.949893000 2.921101000

16 -0.908186000 0.817455000 2.875841000

1 0.186412000 3.213860000 2.261953000

6 4.827012000 -0.586684000 0.792402000

6 3.745904000 -0.213451000 1.645596000

6 6.168340000 -0.493208000 1.274326000

6 4.013022000 0.240270000 2.948020000

6 6.389852000 -0.032492000 2.582690000

6 5.325401000 0.328791000 3.407696000

1 3.183630000 0.519663000 3.591366000

1 7.410232000 0.038829000 2.949970000

1 5.520454000 0.680903000 4.416138000

6 4.567473000 -1.053376000 -0.531032000

6 3.224319000 -1.149759000 -1.022621000

6 5.659916000 -1.428034000 -1.373984000

6 3.014561000 -1.615955000 -2.330713000

6 5.396640000 -1.887650000 -2.674081000

6 4.089155000 -1.979395000 -3.141604000

1 2.001388000 -1.699586000 -2.708630000

1 6.227781000 -2.172619000 -3.313374000

1 3.899613000 -2.338589000 -4.148760000

6 2.158232000 -0.751947000 -0.125196000

6 2.406099000 -0.316537000 1.146383000

6 7.243167000 -0.876049000 0.401024000

6 6.999136000 -1.322084000 -0.861396000

1 7.821036000 -1.609626000 -1.511573000

1 8.261907000 -0.802838000 0.772150000

1 1.579886000 -0.036424000 1.796468000

TS_3-7_

6 2.728370000 4.281025000 2.424737000

6 1.983627000 4.904237000 1.423564000

6 2.662670000 2.891442000 2.609627000

1 3.245718000 2.429240000 3.400481000

6 1.176826000 4.109593000 0.609584000

6 1.858397000 2.095387000 1.798979000

1 1.787265000 1.022172000 1.943652000

6 1.110817000 2.708425000 0.785518000

1 3.362724000 4.881311000 3.069575000

6 -0.332025000 2.901122000 -0.958064000

6 -4.200329000 -0.052854000 -0.514165000

6 -3.350805000 -0.843272000 0.275830000

6 -5.569663000 -0.271649000 -0.438322000

1 -6.239479000 0.331807000 -1.042751000

6 -3.894197000 -1.830954000 1.131282000

6 -6.102518000 -1.257596000 0.411194000

1 -7.176910000 -1.408030000 0.451028000

6 -5.272554000 -2.043182000 1.206394000

1 -5.685595000 -2.800670000 1.864416000

1 -3.783095000 0.717280000 -1.155183000

16 -2.598868000 -2.635920000 1.994901000

7 -1.967140000 -0.747266000 0.327776000

6 -1.411578000 -1.608347000 1.179926000

7 0.264899000 2.069058000 -0.109965000

16 0.123036000 4.589666000 -0.720398000

1 2.031073000 5.979492000 1.283779000

16 -1.423760000 2.304768000 -2.150325000

16 0.269827000 -1.730082000 1.459769000

30 -0.594131000 0.282816000 -0.880123000

16 1.105096000 -2.779963000 -1.220142000

16 0.433523000 -1.274624000 -2.440777000

6 3.758890000 -1.510709000 -0.335140000

6 4.399803000 -1.472648000 -1.701397000

1 3.793937000 -0.870727000 -2.384111000

1 5.398217000 -1.015945000 -1.656004000

1 4.509239000 -2.466418000 -2.143635000

6 4.176529000 -2.294668000 0.785391000

6 5.080989000 -3.318907000 0.898299000

6 5.995216000 -3.918316000 -0.126534000

1 6.992513000 -4.073129000 0.302551000

1 6.103585000 -3.313891000 -1.025830000

1 5.636348000 -4.912256000 -0.430454000

1 5.153874000 -3.772172000 1.886181000

1 3.645044000 -2.053633000 1.705925000

1 3.234990000 -0.593779000 -0.058936000

1 2.397723000 -2.207984000 -0.700323000

7

6 3.155511000 3.889713000 2.432600000

6 2.506784000 4.580405000 1.409154000

6 2.916076000 2.522804000 2.641335000

1 3.427165000 2.007605000 3.448942000

6 1.618823000 3.875630000 0.596822000

6 2.029881000 1.816442000 1.832748000

1 1.824294000 0.763434000 1.995792000

6 1.377702000 2.497170000 0.796685000

1 3.850994000 4.419521000 3.076104000

6 -0.005176000 2.832359000 -0.973814000

6 -4.208546000 0.360102000 -0.609747000

6 -3.486060000 -0.507155000 0.226092000

6 -5.595010000 0.297025000 -0.589427000

1 -6.167793000 0.960866000 -1.229160000

6 -4.172229000 -1.413083000 1.070941000

6 -6.269916000 -0.608673000 0.249624000

1 -7.355119000 -0.636952000 0.245147000

6 -5.567838000 -1.468045000 1.089824000

1 -6.090461000 -2.162477000 1.739345000

1 -3.681171000 1.067704000 -1.241502000

16 -3.013653000 -2.341461000 1.999731000

7 -2.105073000 -0.564729000 0.335347000

6 -1.685735000 -1.467352000 1.223344000

7 0.471645000 1.949746000 -0.100967000

16 0.651417000 4.455767000 -0.758770000

1 2.688987000 5.638556000 1.250868000

16 -1.143650000 2.350735000 -2.174825000

16 -0.043925000 -1.769309000 1.579925000

30 -0.583522000 0.269219000 -0.857508000

16 0.802371000 -2.973888000 -1.070954000

16 0.241486000 -1.453906000 -2.354902000

6 4.036298000 -1.413589000 -0.181609000

6 4.516283000 -1.394655000 -1.603974000

1 3.824032000 -0.825116000 -2.232933000

1 5.495229000 -0.898317000 -1.686553000

1 4.619198000 -2.391373000 -2.036552000

6 4.204579000 -2.406267000 0.784635000

6 4.848996000 -3.641072000 0.735296000

6 5.622755000 -4.243650000 -0.401170000

1 6.282792000 -5.036797000 -0.037420000

1 6.240424000 -3.508055000 -0.924744000

1 4.962572000 -4.702348000 -1.152407000

1 4.780413000 -4.246738000 1.636204000

1 3.749579000 -2.175999000 1.748616000

1 3.550703000 -0.499718000 0.156959000

1 1.974035000 -2.481150000 -0.571554000

TS_7-8_

6 5.583557000 2.107544000 -0.364574000

6 5.687968000 0.805716000 -0.853485000

6 4.389143000 2.564483000 0.213358000

1 4.332985000 3.580040000 0.593093000

6 4.573485000 -0.027020000 -0.756023000

6 3.275258000 1.735763000 0.310491000

1 2.350983000 2.076450000 0.764201000

6 3.364280000 0.428695000 -0.183410000

1 6.439407000 2.772041000 -0.429681000

6 2.730317000 -1.695344000 -0.685670000

6 -2.324328000 -2.536397000 -1.608837000

6 -2.504583000 -1.353336000 -0.872096000

6 -3.428308000 -3.130290000 -2.214595000

1 -3.291319000 -4.044074000 -2.785175000

6 -3.802448000 -0.792290000 -0.768339000

6 -4.708143000 -2.569589000 -2.102224000

1 -5.555006000 -3.049950000 -2.582136000

6 -4.902766000 -1.392065000 -1.376099000

1 -5.891533000 -0.952511000 -1.288516000

1 -1.332746000 -2.967267000 -1.707089000

16 -3.749889000 0.688750000 0.188627000

7 -1.518679000 -0.641292000 -0.217561000

6 -1.997039000 0.455624000 0.466403000

7 2.355746000 -0.525672000 -0.170025000

16 4.395105000 -1.706934000 -1.261138000

1 6.613411000 0.450701000 -1.295390000

16 1.629380000 -3.018061000 -0.734859000

16 -0.964927000 1.654262000 1.121267000

30 0.376847000 -1.086210000 0.305511000

16 -1.564486000 -1.271343000 3.421012000

16 0.237394000 -1.896088000 2.763436000

6 -0.407333000 3.171414000 -0.351369000

6 0.076415000 4.288240000 0.551159000

1 -0.690723000 4.613039000 1.258304000

1 0.941866000 3.962977000 1.138193000

1 0.389871000 5.158358000 -0.039663000

6 -1.509522000 3.338506000 -1.302113000

6 -2.459716000 4.290021000 -1.439482000

6 -2.724488000 5.544121000 -0.657890000

1 -2.800213000 6.405834000 -1.333726000

1 -1.967648000 5.770033000 0.090971000

1 -3.692986000 5.473106000 -0.143969000

1 -3.165927000 4.126979000 -2.253749000

1 -1.570200000 2.521608000 -2.020989000

1 0.421542000 2.631717000 -0.810035000

1 -1.888347000 -0.386176000 2.388374000

8

6 5.725208000 0.242499000 1.128483000

6 5.514016000 -0.005434000 -0.227900000

6 4.675656000 0.121117000 2.052058000

1 4.863809000 0.312522000 3.104155000

6 4.234390000 -0.375077000 -0.641334000

6 3.396422000 -0.246417000 1.642171000

1 2.581187000 -0.344823000 2.351289000

6 3.169691000 -0.495596000 0.281909000

1 6.714808000 0.528198000 1.471318000

6 2.041628000 -1.058725000 -1.606823000

6 -3.333057000 -1.590692000 -1.070791000

6 -2.952635000 -0.389322000 -0.450855000

6 -4.654588000 -1.741509000 -1.464450000

1 -4.965250000 -2.665245000 -1.941586000

6 -3.909839000 0.628213000 -0.234343000

6 -5.599922000 -0.721169000 -1.249124000

1 -6.627543000 -0.868526000 -1.566525000

6 -5.241669000 0.471179000 -0.631305000

1 -5.972846000 1.254447000 -0.461421000

1 -2.610448000 -2.385903000 -1.215677000

16 -3.142703000 1.981804000 0.560206000

7 -1.674716000 -0.077659000 0.008102000

6 -1.619916000 1.115660000 0.549032000

7 1.962219000 -0.864382000 -0.293056000

16 3.657571000 -0.761537000 -2.262028000

1 6.327019000 0.083425000 -0.941468000

16 0.670210000 -1.570766000 -2.508136000

16 -0.179694000 1.829515000 1.277482000

30 -0.062468000 -1.548143000 -0.067323000

16 -1.576894000 -2.405451000 2.861310000

16 -0.650095000 -3.407189000 1.222996000

6 0.693921000 2.577947000 -0.236306000

6 1.984266000 3.210250000 0.303897000

1 1.782752000 4.051510000 0.970732000

1 2.592380000 2.474223000 0.836743000

1 2.567898000 3.586339000 -0.541769000

6 -0.169566000 3.494733000 -1.066653000

6 -0.714416000 4.675043000 -0.738042000

6 -0.677556000 5.399697000 0.577013000

1 -0.059967000 6.304547000 0.502949000

1 -0.294043000 4.786187000 1.393140000

1 -1.684586000 5.734852000 0.852639000

1 -1.270503000 5.184721000 -1.524888000

1 -0.342693000 3.135542000 -2.079675000

1 0.961297000 1.708451000 -0.839271000

1 -0.528286000 -2.378293000 3.714590000

6

6 -4.021334000 4.728090000 0.553506000

6 -5.192385000 4.053373000 0.209990000

6 -2.781210000 4.070381000 0.545995000

1 -1.879715000 4.613182000 0.812382000

6 -5.096177000 2.707203000 -0.141698000

6 -2.683370000 2.726722000 0.196746000

1 -1.725094000 2.216667000 0.190982000

6 -3.851673000 2.037673000 -0.150700000

1 -4.072521000 5.776944000 0.828954000

6 -5.178096000 0.315111000 -0.806943000

6 -2.427154000 -2.902362000 2.723928000

6 -1.563378000 -2.877057000 1.622173000

6 -2.007495000 -3.543228000 3.885478000

1 -2.667208000 -3.570638000 4.747183000

6 -0.295704000 -3.496349000 1.705039000

6 -0.746161000 -4.154594000 3.960603000

1 -0.441051000 -4.648094000 4.878023000

6 0.123068000 -4.137654000 2.870612000

1 1.098007000 -4.610619000 2.929315000

1 -3.402028000 -2.429790000 2.659371000

16 0.559640000 -3.305731000 0.174156000

7 -1.817307000 -2.285554000 0.392016000

6 -0.816885000 -2.421295000 -0.470879000

7 -3.938725000 0.702816000 -0.525839000

16 -6.375161000 1.590936000 -0.624811000

1 -6.150491000 4.563147000 0.214934000

16 -5.486434000 -1.315821000 -1.302563000

16 1.356847000 2.173575000 1.133173000

30 -3.066979000 -1.188624000 -0.934171000

16 1.382512000 4.150604000 0.375750000

16 -0.951676000 -1.752207000 -2.063336000

1 2.218093000 4.716484000 1.277357000

6 4.760077000 -0.098296000 -1.033733000

6 3.397772000 -0.249197000 -1.429760000

6 5.778497000 -0.825909000 -1.721162000

6 3.075025000 -1.113362000 -2.489942000

6 5.411502000 -1.678080000 -2.776180000

6 4.076951000 -1.819563000 -3.153419000

1 2.035157000 -1.224088000 -2.784324000

1 6.186471000 -2.232110000 -3.299510000

1 3.815309000 -2.484558000 -3.971115000

6 5.098089000 0.771257000 0.046596000

6 4.080130000 1.498669000 0.743569000

6 6.466064000 0.905683000 0.440815000

6 4.456119000 2.318658000 1.818518000

6 6.790066000 1.749343000 1.514957000

6 5.792931000 2.440825000 2.196071000

1 3.689005000 2.849828000 2.371234000

1 7.829919000 1.851587000 1.813625000

1 6.055620000 3.081591000 3.032830000

6 2.707175000 1.342251000 0.297351000

6 2.393066000 0.504224000 -0.737786000

6 7.144008000 -0.660752000 -1.305492000

6 7.470107000 0.165061000 -0.274024000

1 8.506564000 0.279117000 0.032536000

1 7.915875000 -1.214290000 -1.833670000

1 1.360270000 0.400875000 -1.057458000

9

6 3.800096000 -3.535955000 2.154139000

6 4.749929000 -2.754020000 1.497881000

6 2.429388000 -3.263170000 2.024825000

1 1.707147000 -3.888146000 2.540637000

6 4.298741000 -1.694717000 0.710945000

6 1.976226000 -2.206361000 1.240976000

1 0.915904000 -1.998642000 1.137450000

6 2.920200000 -1.413787000 0.576807000

1 4.128097000 -4.367070000 2.770691000

6 3.743419000 0.238828000 -0.745922000

6 0.279125000 3.624006000 1.791934000

6 -0.552140000 3.063809000 0.814217000

6 -0.276632000 4.504760000 2.714627000

1 0.356477000 4.947038000 3.477521000

6 -1.924762000 3.398707000 0.779112000

6 -1.641253000 4.831310000 2.673995000

1 -2.051422000 5.521584000 3.404433000

6 -2.479850000 4.281655000 1.705163000

1 -3.534344000 4.536097000 1.673482000

1 1.333976000 3.370110000 1.816766000

16 -2.707769000 2.549748000 -0.554309000

7 -0.167937000 2.172218000 -0.178245000

6 -1.167658000 1.808680000 -0.972020000

7 2.649801000 -0.325736000 -0.244144000

16 5.236420000 -0.538387000 -0.236669000

1 5.809726000 -2.965785000 1.596692000

16 3.610219000 1.609899000 -1.795558000

16 -1.909063000 -2.992285000 0.739275000

30 1.310796000 1.019358000 -1.191134000

16 -1.875805000 -3.099565000 -1.344975000

16 -0.877565000 0.714248000 -2.283587000

6 -3.717597000 -2.679659000 1.166803000

6 -4.580896000 -3.893484000 0.826057000

1 -4.502881000 -4.161169000 -0.230036000

1 -4.256040000 -4.756515000 1.413238000

1 -5.632253000 -3.692680000 1.057363000

6 -4.129572000 -1.314814000 0.687364000

6 -4.968820000 -0.914266000 -0.279564000

6 -5.824182000 -1.683409000 -1.246413000

1 -6.882304000 -1.425485000 -1.106780000

1 -5.723646000 -2.763568000 -1.168738000

1 -5.570451000 -1.397559000 -2.274592000

1 -5.054587000 0.167314000 -0.392762000

1 -3.615356000 -0.519586000 1.226555000

1 -3.619202000 -2.621165000 2.260346000

1 -1.731332000 -1.778087000 -1.623903000

10

16 -0.832034000 -1.844512000 -2.042856000

16 -2.329103000 -2.068037000 -0.548607000

6 2.506599000 0.814394000 -0.240777000

6 1.604228000 1.312908000 -1.227401000

6 3.529618000 1.667557000 0.274715000

6 1.736393000 2.636858000 -1.678305000

6 3.623538000 2.984913000 -0.204460000

6 2.737545000 3.461757000 -1.169293000

1 1.047805000 3.011559000 -2.430774000

1 4.402252000 3.634024000 0.187232000

1 2.828802000 4.483061000 -1.527143000

6 2.385972000 -0.530946000 0.221618000

6 1.364768000 -1.393390000 -0.293634000

6 3.302223000 -1.021275000 1.203819000

6 1.298479000 -2.714476000 0.174828000

6 3.187217000 -2.348559000 1.645861000

6 2.198640000 -3.182002000 1.131695000

1 0.539485000 -3.378024000 -0.222485000

1 3.883948000 -2.720339000 2.392451000

1 2.125161000 -4.209261000 1.476344000

6 0.449216000 -0.849295000 -1.281407000

6 0.577094000 0.441880000 -1.719703000

6 4.429040000 1.144567000 1.265841000

6 4.319155000 -0.138120000 1.707091000

1 5.007457000 -0.522844000 2.455078000

1 5.204992000 1.797672000 1.656226000

1 -0.117643000 0.824857000 -2.461321000

6 -5.826290000 1.455310000 1.508290000

1 -6.749521000 1.564920000 0.927098000

1 -5.543703000 0.402237000 1.484589000

1 -6.075358000 1.727513000 2.542656000

6 -4.762714000 2.364991000 0.961363000

6 -3.493387000 2.084491000 0.631174000

6 -2.753843000 0.768257000 0.740710000

1 -5.081567000 3.396975000 0.812414000

1 -2.901734000 2.897059000 0.213550000

6 -2.701492000 0.149342000 2.139655000

1 -2.204866000 0.845803000 2.824398000

1 -3.696351000 -0.064346000 2.534802000

1 -2.126374000 -0.780667000 2.126020000

16 -3.531582000 -0.368104000 -0.541802000

1 -1.731296000 0.925252000 0.391013000

11

16 0.843671000 -0.520365000 -1.486800000

16 0.000013000 -1.760989000 0.000226000

6 4.943046000 0.868856000 0.160030000

6 3.806683000 1.731493000 0.165520000

6 6.191865000 1.344884000 0.663971000

6 3.932983000 3.037464000 0.666903000

6 6.271763000 2.658352000 1.156031000

6 5.155320000 3.493005000 1.156772000

1 3.064171000 3.690014000 0.670403000

1 7.221965000 3.020539000 1.539609000

1 5.238925000 4.504900000 1.542143000

6 4.828220000 -0.459832000 -0.349361000

6 3.579806000 -0.942944000 -0.859090000

6 5.976185000 -1.312261000 -0.354311000

6 3.518743000 -2.250903000 -1.363450000

6 5.860199000 -2.615011000 -0.863774000

6 4.645096000 -3.072911000 -1.363599000

1 2.581501000 -2.619922000 -1.763232000

1 6.733113000 -3.262352000 -0.866405000

1 4.569507000 -4.081269000 -1.759806000

6 2.439880000 -0.043454000 -0.827313000

6 2.562126000 1.228618000 -0.337432000

6 7.324131000 0.460356000 0.648122000

6 7.219123000 -0.806301000 0.161828000

1 8.083256000 -1.465452000 0.153992000

1 8.271917000 0.826420000 1.033836000

1 1.695666000 1.882439000 -0.323725000

16 -0.843744000 -0.520266000 1.487101000

6 -4.828225000 -0.459813000 0.349261000

6 -5.976183000 -1.312243000 0.354106000

6 -3.579852000 -0.942930000 0.859107000

6 -5.860236000 -2.615003000 0.863565000

6 -3.518838000 -2.250880000 1.363488000

6 -4.645188000 -3.072899000 1.363519000

1 -6.733148000 -3.262348000 0.866104000

1 -2.581634000 -2.619881000 1.763370000

1 -4.569638000 -4.081247000 1.759756000

6 -4.943018000 0.868880000 -0.160108000

6 -3.806676000 1.731535000 -0.165437000

6 -6.191788000 1.344893000 -0.664188000

6 -3.932940000 3.037521000 -0.666793000

6 -6.271658000 2.658384000 -1.156198000

6 -5.155231000 3.493060000 -1.156779000

1 -3.064136000 3.690082000 -0.670180000

1 -7.221818000 3.020567000 -1.539883000

1 -5.238815000 4.504967000 -1.542122000

6 -7.324024000 0.460328000 -0.648541000

6 -7.219083000 -0.806288000 -0.162118000

6 -2.562158000 1.228642000 0.337575000

6 -2.439936000 -0.043419000 0.827443000

1 -8.271770000 0.826389000 -1.034354000

1 -8.083205000 -1.465455000 -0.154372000

1 -1.695701000 1.882469000 0.323957000

12

16 1.719249000 -0.926426000 -0.606285000

16 0.029961000 -1.433920000 0.539920000

6 2.469567000 0.609797000 0.176131000

6 -5.218976000 -0.449175000 0.117170000

1 -5.754219000 -1.152505000 -0.531551000

1 -4.452113000 -1.010863000 0.651590000

1 -5.952599000 -0.076462000 0.844544000

6 -4.660349000 0.680644000 -0.700701000

6 -3.420432000 1.192428000 -0.708050000

6 -2.224231000 0.794379000 0.131106000

1 -5.373246000 1.140371000 -1.385715000

1 -3.222650000 1.993388000 -1.417946000

6 -2.426407000 0.856720000 1.647315000

1 -2.676468000 1.882654000 1.940212000

1 -3.232811000 0.201976000 1.982293000

1 -1.510885000 0.565772000 2.170979000

16 -1.721415000 -0.904981000 -0.483683000

6 3.660984000 0.898148000 -0.695365000

6 4.929549000 0.525298000 -0.475007000

6 1.506309000 1.791197000 0.254623000

1 0.642165000 1.551292000 0.878924000

1 2.015570000 2.652770000 0.701154000

1 1.153062000 2.078223000 -0.741060000

6 5.471886000 -0.265894000 0.678996000

1 5.948208000 -1.186125000 0.319072000

1 6.248249000 0.301489000 1.207716000

1 4.704909000 -0.551954000 1.401794000

1 3.436712000 1.454559000 -1.604519000

1 5.669307000 0.826508000 -1.216655000

1 2.781319000 0.318207000 1.181302000

1 -1.396607000 1.454685000 -0.138682000

**References**

(1) Manna, R.; Srivastava, S. K. Fabrication of Functionalized Graphene Filled Carboxylated Nitrile Rubber Nanocomposites as Flexible Dielectric Material. 10.

(2) Liu, X.; Sun, D.; Wang, L.; Guo, B. Sodium Humate Functionalized Graphene and Its Unique Reinforcement Effects for Rubber. *Ind. Eng. Chem. Res.* **2013**, *52* (41), 14592–14600. https://doi.org/10.1021/ie402318r.

(3) Yang, D.; Kong, X.; Ni, Y.; Ruan, M.; Huang, S.; Shao, P.; Guo, W.; Zhang, L. Improved Mechanical and Electrochemical Properties of XNBR Dielectric Elastomer Actuator by Poly(Dopamine) Functionalized Graphene Nano-Sheets. *Polymers* **2019**, *11* (2), 218. https://doi.org/10.3390/polym11020218.

(4) Kang, H.; Zuo, K.; Wang, Z.; Zhang, L.; Liu, L.; Guo, B. Using a Green Method to Develop Graphene Oxide/Elastomers Nanocomposites with Combination of High Barrier and Mechanical Performance. *Compos. Sci. Technol.* **2014**, *92*, 1–8. https://doi.org/10.1016/j.compscitech.2013.12.004.

(5) Thomas, P. C.; Thomas, S. P.; George, G.; Thomas, S.; Kuruvilla, J. Impact of Filler Geometry and Surface Chemistry on the Degree of Reinforcement and Thermal Stability of Nitrile Rubber Nanocomposites. *J. Polym. Res.* **2011**, *18* (6), 2367–2378. https://doi.org/10.1007/s10965-011-9651-1.

(6) Hernandez, Y., Lotya, M.; Rickard, D.; Bergin, S. D.; Coleman, J. N. Measurement of Multicomponenet Solubility Parameters for Graphene Facilitates Solvent Discovery. *Langmuir* **2010**, *26(5)*, 3208-3213. https://doi.org/10.1021/la903188a.
